# Interactions of human milk oligosaccharides with the immune system

**DOI:** 10.3389/fimmu.2024.1523829

**Published:** 2025-01-14

**Authors:** Alanna S. Slater, Rita M. Hickey, Gavin P. Davey

**Affiliations:** ^1^ Teagasc Food Research Centre, Moorepark, Fermoy, Ireland; ^2^ School of Biochemistry and Immunology, Trinity Biomedical Sciences Institute, Trinity College Dublin, Dublin, Ireland

**Keywords:** human milk oligosaccharides (HMOs), immunomodulation, immune cell receptors, gut-associated immune responses, autoimmune and inflammatory diseases

## Abstract

Human milk oligosaccharides (HMOs) are abundant, diverse and complex sugars present in human breast milk. HMOs are well-characterized barriers to microbial infection and by modulating the human microbiome they are also thought to be nutritionally beneficial to the infant. The structural variety of over 200 HMOs, including neutral, fucosylated and sialylated forms, allows them to interact with the immune system in various ways. Clinically, HMOs impact allergic diseases, reducing autoimmune and inflammatory responses, and offer beneficial support to the preterm infant immune health. This review examines the HMO composition and associated immunomodulatory effects, including interactions with immune cell receptors and gut-associated immune responses. These immunomodulatory properties highlight the potential for HMO use in early stage immune development and for use as novel immunotherapeutics. HMO research is rapidly evolving and promises innovative treatments for immune-related conditions and improved health outcomes.

## 1 Introduction

**Figure 1:**
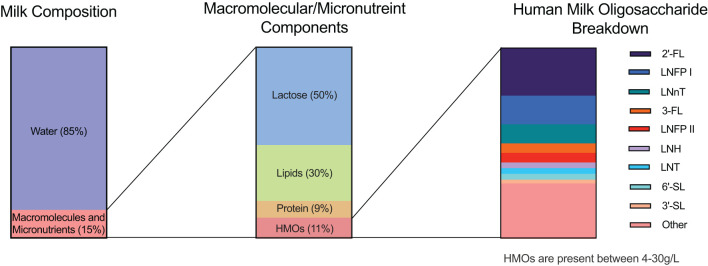
Human milk composition. Breast milk is composed of approximately 85% water, which provides hydration to the infant. The remaining 15% comprises an assortment of macromolecules and essential micronutrients that play crucial roles in infant development. Within the macromolecule fraction, lactose accounts for approximately 50%, serving as the primary carbohydrate source in breast milk. Lipids, encompassing essential fatty acids and other vital compounds, constitute ~30% of the macromolecules, providing energy and aiding in the absorption of fat-soluble vitamins. Proteins contribute ~9%, delivering essential amino acids necessary for growth and development. Human milk oligosaccharides (HMOs) make up the remaining ~11% of the macromolecules, representing a diverse array of complex carbohydrates exclusive to human milk. Of the HMOs present, three dominant oligosaccharides are highlighted. The first is 2’-fucosyllactose (2’FL), followed by Lacto-*N*-fucopentaose I (LNFP I) and Lacto-*N*-neotetraose (LNnT). These three HMOs collectively constitute approximately 50% of the total HMOs secreted in breast milk. The remaining 50% encompasses other HMOs, including 3-fucosyllactose (3-FL), Lacto-*N*-fucopentaose II (LNFP II), Lacto-*N*-hexaose (LNH), Lacto-*N*-tetraose (LNT), 3’-sialyllactose (3’SL), 6’-sialyllactose (6’SL), and various additional structures. More than 200 HMOs have currently been identified to date.

Breast milk is often described as the gold standard when it comes to infant nutrition, containing a high content of protein, fat, carbohydrates, vitamins, and minerals. It is regarded as the most beneficial source of nutrients for infant growth and development. As well as transferring maternal antibodies in the form of secretory IgA to the infant (1), providing passive immunity to the infant, it is also a source of other immune compounds that help regulate the naïve and developing immune system in the infant. One of the most important of these components is the Human Milk Oligosaccharide (HMO) fraction found in human milk, which is a diverse group of complex sugars. HMOs are the third most abundant component of breast milk, behind only lactose and fat (2) (**Figure 1**). The consumption of HMOs by infants has been shown to have a number of beneficial effects on the developing gut microbiome and immune system, as well as on the overall health of the infant (3, 4). HMOs were originally described when discovered in the 1900s as ‘bifidus factor’ due to the understanding that HMOs promoted the growth of beneficial bacteria and dampened the growth of pathogenic bacteria (5). However it is now understood that HMOs play an even deeper role in the development of the infant immune system.

HMOs offer a multitude of benefits for breastfed infants: 1. HMOs serve as prebiotics, acting as metabolic substrates for beneficial bacteria in the infant gut microbiota (6). This confers a growth advantage to a subset of microbes, fostering a healthy gut microbiome which is vital for the infant, especially in the first year of life. 2. HMOs also function as anti-adhesives and antimicrobials by acting as soluble glycan receptor decoys impeding the attachment of pathogens to mucosal surfaces in the infant (7). This hinders microbial colonization and potential infection due to the blocking of the microbial glycome from interacting with the glycan surface receptors at the epithelial surface. HMOs possess the ability to modulate the immune response by influencing the production of various cytokines, including inflammatory TNF-α, IFN-γ, IL-1ra, IL-1β, and pleiotropic IL-6 (8). IL-6 is a multi-functional cytokine that can act as pro-inflammatory or anti-inflammatory depending on the cellular context and signaling pathways involved (9). It has been referred to in this review in both a pro and anti-inflammatory manor. The capacity for HMOs to regulate cytokine production within the body holds promise for enhancing immune function, mitigating inflammatory processes, and promoting overall physiological balance. 3. HMOs reduce selectin-mediated cell–cell interactions within the immune system (10). This reduces leukocyte rolling on activated endothelial cells, minimizes mucosal leukocyte infiltration and activation and subsequently influences immune cell dynamics and reduces inflammation.

**Figure 2:**
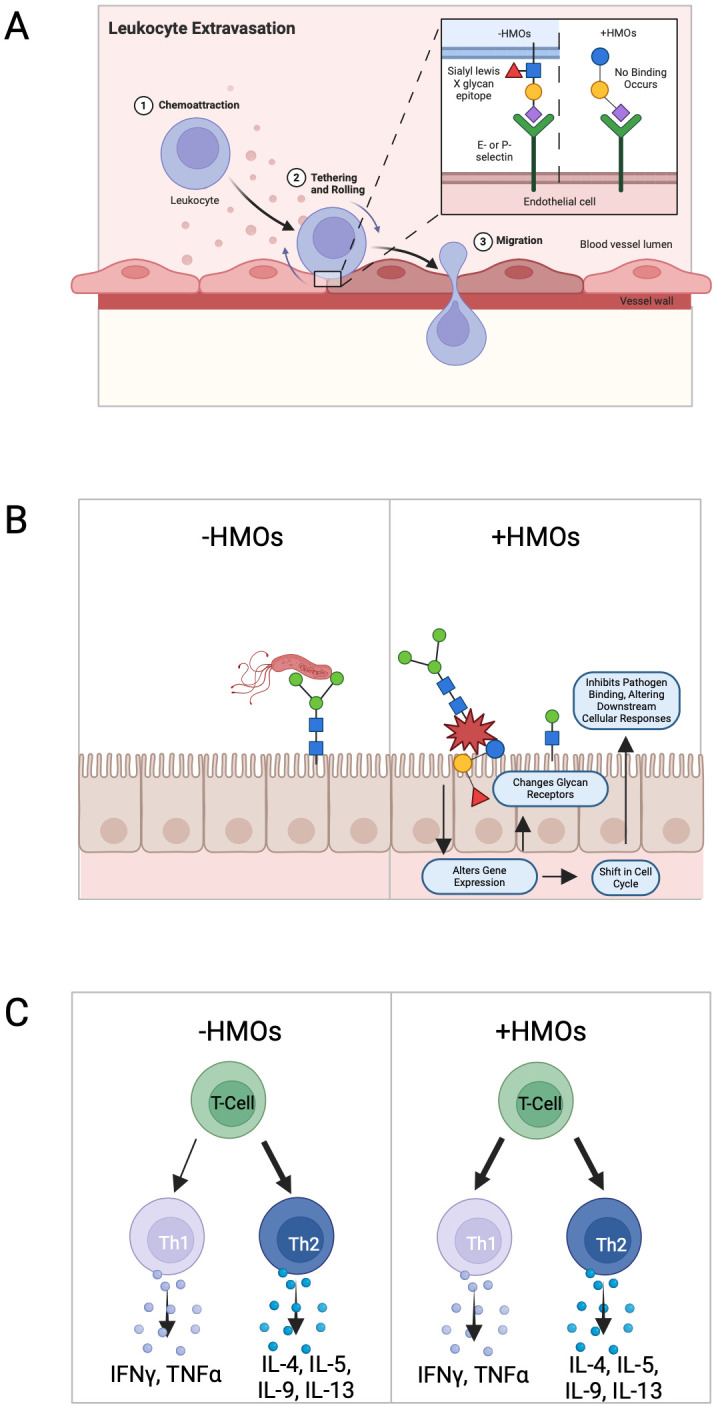
Known effects of human milk oligosaccharide on the immune response. **(A)** HMOs reduce selectin-mediated cell–cell interactions within the immune system. This reduction in leukocyte rolling on activated endothelial cells can potentially minimize mucosal leukocyte infiltration and activation, thereby influencing immune cell dynamics. **(B)** HMOs directly influence intestinal epithelial cells, modulating the expression of genes involved in various cellular processes leading to alterations in cell surface glycans and other cellular responses, thereby impacting gut health and functionality. **(C)** HMOs possess the ability to modulate the production of cytokines by lymphocytes, promoting a balanced Th1/Th2 immune response.

Numerous comprehensive scientific reviews have extensively covered the structural composition (11–13) and other biological roles (6, 14) attributed to HMOs in human breast milk. In the current review, our emphasis is directed towards elucidating the intricate interactions of HMOs with the circulating immune system, as well as exploring the indirect effects of HMOs on the maturation and development of the infant immune system (summarized in **Figure 2**). Moreover, we focus on various mechanisms of action employed by the immune system in response to HMOs presentation, highlighting new mechanisms in which HMOs exert a profound impact on immune function.

## 2 Composition of human milk oligosaccharides and immunomodulation

**Figure 3:**
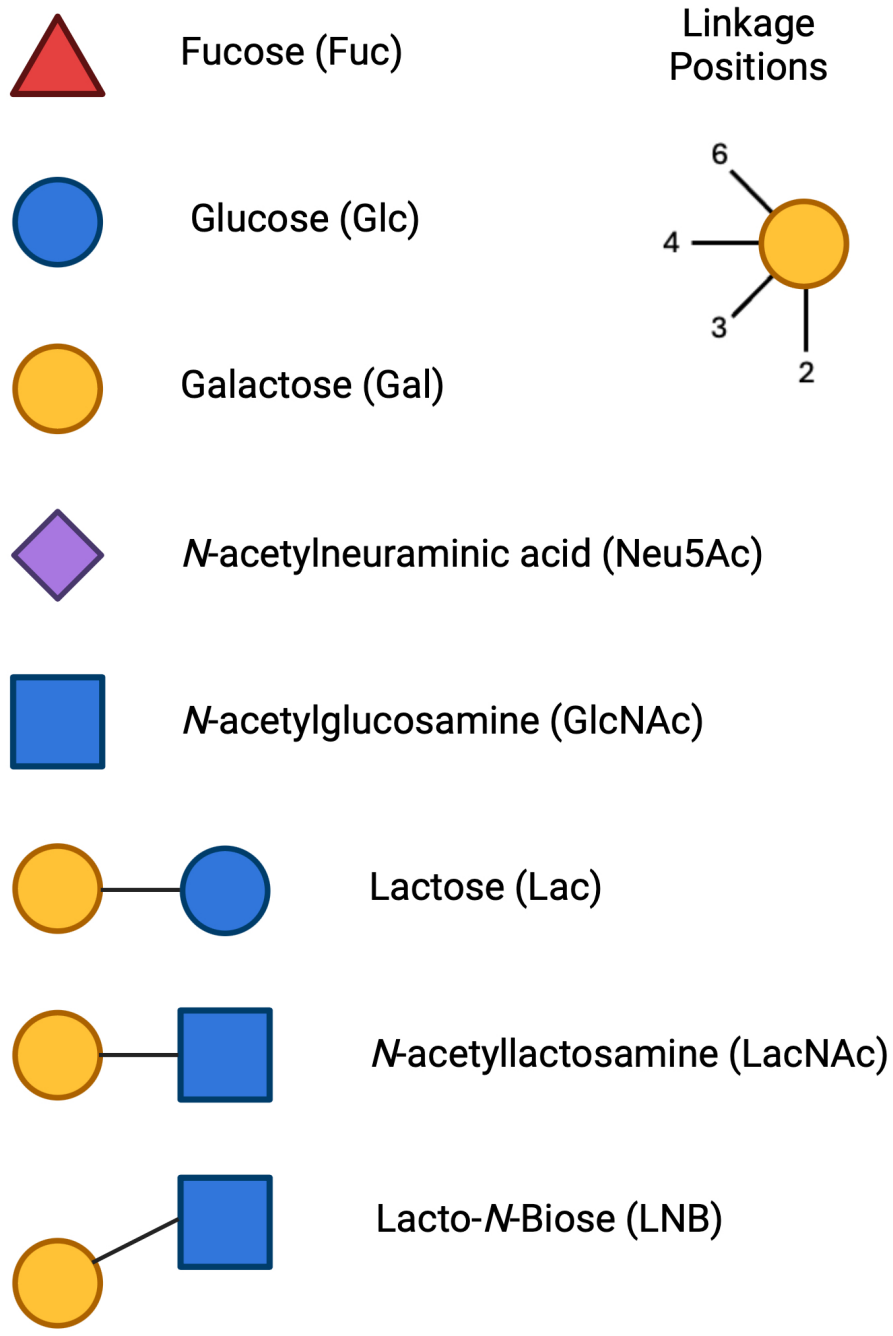
Monosaccharide units and linkage position for HMO formation: The symbols for the main HMO units include Glucose (Glc), Symbol, blue circle; Galactose (Gal), Symbol, yellow circle; *N*-Acetylglucosamine (GlcNAc), Symbol, blue square; Fucose (Fuc), Symbol, red triangle; Sialic Acid (*N*-Acetylneuraminic Acid, Neu5Ac), Symbol, purple diamond. The 3 cores that HMOs can extend from are Lactose, *N*-acetyllactosamine and Lacto-*N*-Biose. Each unit can be linked in a 2- 3- 4- or 6- linkage position.

HMOs are generally composed of five different sugar units, glucose (Glc), galactose (Gal), *N*-acetylglucosamine (GlcNAc), fucose (Fuc) and sialic acid (Sia) primarily in the form of NeuAc (**Table 1**). More than 200 different HMO structures have been identified in human milk to date, and the composition of HMOs can vary greatly between individual lactating mothers (15). HMOs can generally be further divided into subclasses based on their chemical structure (**Figure 3**).

**Table 1:** Composition of human milk oligosaccharides.

Name	Shorthand	1-Letter Symbol
Galactose	Gal	A
Glucose	Glc	G
*N*-acetylglucosamine	GlcNAc	GN
Fucose	Fuc	F
*N*-acetyl-neuraminic acid (Sialic Acid)	NeuAc	NN

**Figure 4:**
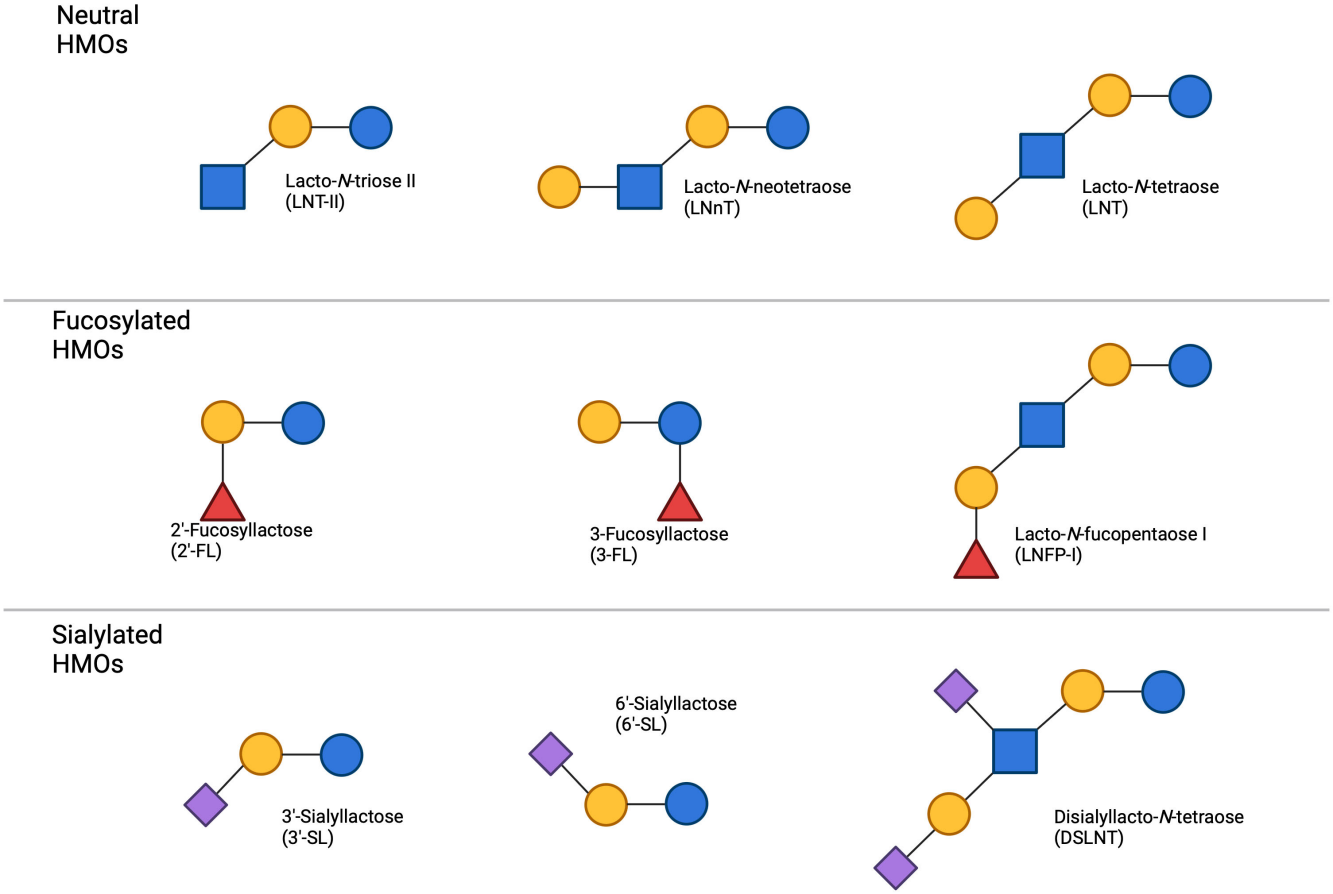
Common HMOs found in breast milk the primary types of HMOs categorized by their structural characteristics. Neutral HMOs, including lacto-*N*-tetraose II (LNT-II), lacto-*N*-neotetraose (LNnT), and lacto-*N*-tetraose (LNT), are depicted on top. The fucosylated HMOs, such as 2’-fucosyllactose (2’-FL), 3-fucosyllactose (3-FL), and lacto-*N*-fucopentaose I (LNFP-I), occupy the centre panel. On the bottom, sialylated HMOs, which include 3’-sialyllactose (3’-SL), 6’-sialyllactose (6’-SL), and disialyllacto-*N*-tetraose (DSLNT), are shown. The figure highlights the structural diversity of HMOs present in breast milk, demonstrating the variation between neutral, fucosylated, and sialylated oligosaccharides.

The structure of HMOs are shaped by the specific sugar units used for their formation, their sequence of incorporation, and the distinct acceptor molecule to which the sugars attach. HMOs are generally classified into three categories: Neutral (core) HMOs, sialylated HMOs, and fucosylated HMOs (**Figure 4**). Neutral HMOs refer to HMOs that do not carry a charge and are neither acidic nor basic, examples of neutral HMOs include lacto-*N*-tetraose (LNT), lacto-*N*-neotetraose (LNnT), lacto-*N*-fucopentaose IV (LNFP-IV) and lacto-*N*-hexaose (LNH). Sialylated HMOs are a subclass of HMOs that have a sialyl group attached to them. This group includes various configurations such as 3’-sialyllactose (3’-SL) and 6’-sialyllactose (6’-SL). In sialylated HMOs, the sialic acid can be added to the lactose, lacto-*N*-biose (LNB), or *N*-acetyllactosamine (LacNAc) residues by α-bonds (16). Fucosylated HMOs are another subclass of HMOs that have a fucosyl group attached to them. This group consists of structures such as 2’-fucosyllactose (2’-FL) and 3-fucosyllactose (3-FL). Fucosylated HMOs, like their sialylated counterparts, can have the fucose added to the lactose, LNB, or LacNAc residues by α-bonds (17). The relative abundance of these HMOs vary from woman to woman with 2’-FL often being the most abundant, making up approximately 30% of total HMOs (18), followed by other key oligosaccharides such as LNT, 3-FL, and 6’-SL (19). Fucosylated HMOs account for 35-50% of all HMOs, with sialylated representing 12-14% and neutral HMOs making up the remaining 42-55% (20) (**Figure 1**).

The variety of HMO structures is generated by multiple glycosyltransferase enzymes and unique combinations of sugar units, enabling a broad spectrum of HMOs to be formed. The activity of these enzymes can be influenced by the respective gene expression in the individual mother; thus the maternal genotype may predetermine the types and amounts of HMOs that a woman can produce (21, 22). Several factors, such as diet (23), age (24), stage of lactation, dictated by various hormones (25) and environmental factors, can influence the expression and activity of glycosyltransferases and the availability of nucleotide sugars thereby influencing infant immune development.

The terms “Secretor positive” and “Lewis positive” refer to differences in individuals based on their blood group antigens and the presence of certain enzymes involved in the synthesis of HMOs. Secretor mothers express the FUT2 gene, which codes for the enzyme α-1,2-fucosyltransferase (21). This enzyme is responsible for the synthesis of HMOs such as 2’-FL or lacto-*N*-fucopentaose I (LNFP-I) by attaching a fucose to lactose or LNT respectively. Lewis positive mothers, on the other hand, express the FUT3 gene, which codes for the enzyme α-1,3-fucosyltransferase (22). This enzyme is also responsible for the synthesis of fucosylated HMOs, synthesising lacto-difucohexaose I (LDFH-I) and lacto-di-fucotetraose (LDFT) from LNFP-I and 2’-FL, respectively (26). Other non-Lewis+ and non-Secretor+ dependent genes expressing enzymes with α-1,3-fucosyltransferase activity are also present, responsible for generating trace amounts of these fucosylated HMOs in -/- mothers. It is worth noting that not all individuals have the same expression levels of FUT2 and FUT3 genes, which affects the quantity of HMOs that can be produced by a mother. The production of HMOs is also regulated by multiple other genes including those that code for the enzymes involved in lactose synthesis, the process by which HMOs are formed, and those that regulate the expression of these enzymes, such as transcription factors and signalling molecules.

Genetic polymorphisms, notably in fucosyltransferase and sialyltransferase genes, guide the abundance and diversity of HMO structures, which in turn modulate neonatal exposure to immunologically active glycan motifs. For example, one study found that a Single Nucleotide Polymorphism (SNP) in FUT2 was associated with lower levels of 2’-FL in human milk (27). This study also found that a SNP in the gene that codes for GCNT3 was associated with increased levels of fucosyllacto-*N*-hexaose (FLNH) in human milk. Understanding these maternal determinants is crucial for tailoring potential HMO fortified diets to enhance HMO-mediated immunological benefits, ultimately improving infant health outcomes. This would allow non-breastfed infants to obtain the same immunological benefits as their breast-fed counterparts.

Several HMOs have been produced at a large scale and received regulatory approval for inclusion in infant formulas. The most common HMOs commercially available are 2’-FL and LNnT, both of which have been approved by the U.S. Food and Drug Administration (FDA), the European Food Safety Authority (EFSA) (28), and other regulatory bodies. These HMOs are synthesized using microbial fermentation or enzymatic processes (29). In the EU, HMOs are regulated as Novel Foods, whereas in the United States, they are categorized as “Generally Recognized as Safe (GRAS)” substances. Indeed, 2’-FL and LNnT are now commonly added to many commercial infant formulas (30) to more closely mimic the benefits of natural breast milk (31, 32), contributing to the overall health and development of formula-fed infants. Infant formula containing 2’-FL and LNnT is safe, well-tolerated, and promotes growth appropriate to infant age (33). As of December 2023 HMOs approved in both the EU and the United States include: 2’-FL (34), 3-FL (35), LNT (36), LNnT (37), 3’-SL (38) and 6’-SL (39), a 2’-FL/difucosyllactose (DFL) mixture (40), and an LNFP-I/2’-FL mixture (41). The inclusion of these HMOs in infant formulas is a significant advancement in paediatric nutrition, bridging the gap between formula and breast milk, subsequently improving growth and development in infants.

## 3 Immunological impacts of human milk oligosaccharides

**Figure 5:**
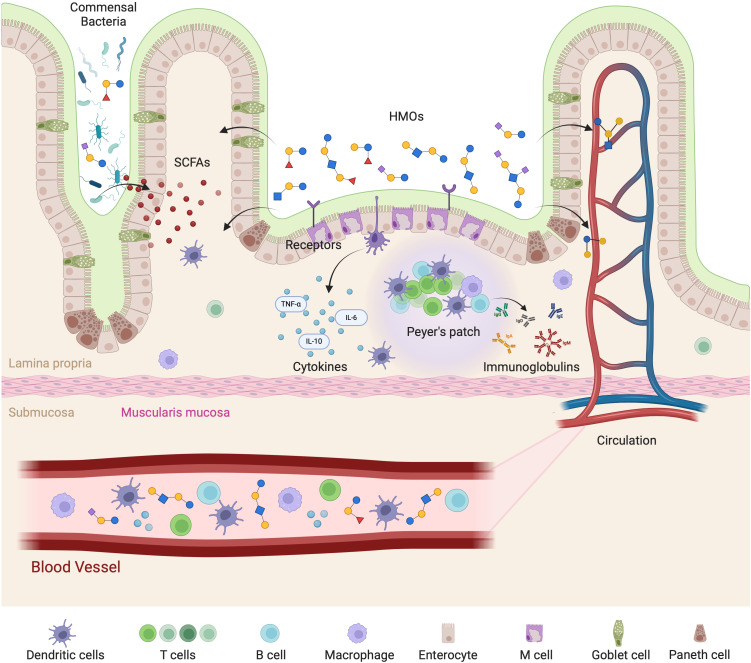
Mechanistic representation of HMOs interaction with the gut-immune axis. HMOs play a vital role in modulating gut immunity and systemic immune responses. HMOs influence gut-associated lymphoid tissue (GALT) by interacting with receptors on enterocytes and immune cells, leading to the release of cytokines such as TNF-α, IL-6, and IL-10. HMOs also promote the growth of beneficial commensal bacteria, which produce short-chain fatty acids (SCFAs) that support mucosal immunity and gut barrier integrity. Additionally, HMOs are involved in the activation of Peyer’s patches, stimulating B cells to produce immunoglobulins that contribute to immune defense. The effects extend beyond the gut, as HMOs and immune-modulating factors enter circulation to influence systemic immune responses. The interplay among epithelial cells, immune cells (dendritic cells, T cells, B cells, and macrophages), and microbial metabolites in maintaining immune homeostasis is highlighted.

HMOs provide small nutritive value (42) and were therefore originally thought to be synthesized for the benefit of the developing gut microbiome. They were first discovered and are still widely recognized as prebiotics, orchestrating a cascade of interactions that influence the host immune system. On arrival in the colon, these intact complex carbohydrates act as a selective substrate for beneficial gut microbiota, fostering an intestinal environment that facilitates the development of a robust immune system. There is now evidence of HMOs present in infant blood, urine and faeces (43–46) which allows us to assume that HMOs interact with cells of the circulating immune system and not just those present at the gut barrier and Gut-Associated Lymphoid Tissue (GALT) (**Figure 5**). In-depth reviews detailing how HMOs influence intestinal epithelial cells, microbiota, and mucosal immune responses, including GALT macrophages and dendritic cells can be found widely in the literature (47–49). Similarly, other publications discuss the ways in which HMOs promote immune tolerance and defense against pathogens specifically in the gut (50, 51). Many studies have been conducted on how HMOs impact the gut microbiota, and in turn the immune system, but the mechanistic interaction between HMOs and circulating immune cells is still largely unknown. Studies to date that have analyzed the effect of HMOs on immune cells have largely focused on the effect of pooled HMOs isolated from breastmilk, which contains unknown amounts and types of HMOs (52–55). However, an in-depth knowledge on the specific activity of individual HMOs on immune cells is lacking. Due to the variability of types and quantities of HMOs among individual donors many studies often convey variable results.

### 3.1 Receptors for human milk oligosaccharides and immune system interaction

**Figure 6:**
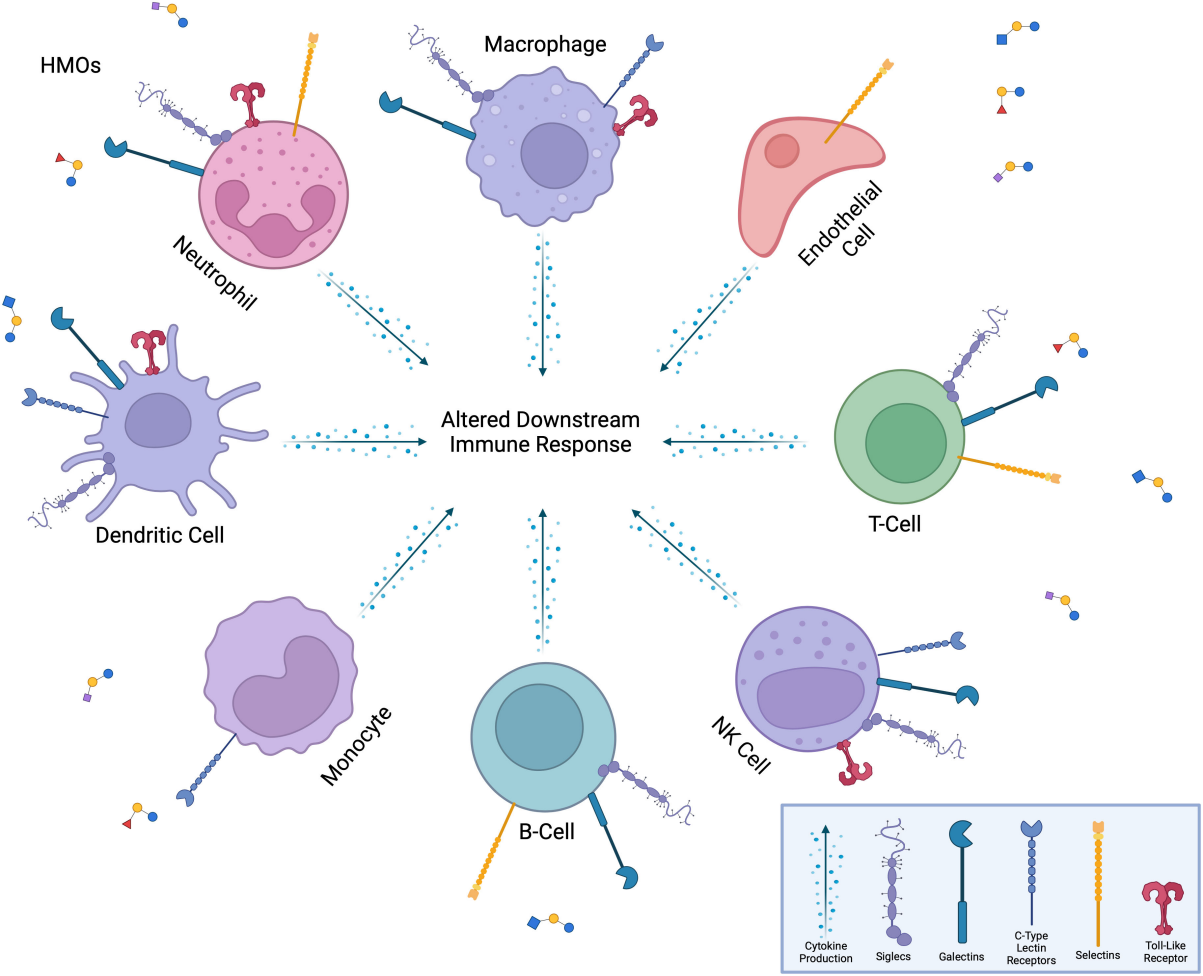
Interaction of HMOs with immune cells and receptors. HMOs influence a diverse range of immune cells, including neutrophils, macrophages, dendritic cells, monocytes, B-cells, T-cells, NK cells, and endothelial cells. HMOs bind to various immune cell surface receptors, including Siglecs, galectins, C-type lectin receptors, selectins, and toll-like receptors, modulating immune cell functions. These interactions lead to altered downstream immune responses, such as changes in cytokine production, cell activation, or metabolic activity, thereby shaping the overall immune response. The versatility of HMOs in modulating both innate and adaptive immune pathways is highlighted.

The impact of HMOs on the immune system may be linked to their interaction with specific glycan-binding receptors known as lectins that are expressed on the surface of intestinal epithelial cells. The interaction of HMOs with specific receptors plays a critical role in shaping the development and function of the infant immune system (**Figure 6**).

#### 3.1.1 Galectins

Galectins, a class of glycan-binding proteins, play crucial roles in intracellular signaling, intercellular communication, cellular proliferation, and survival. These endogenous lectins are expressed and secreted by various cell types, including immune cells, tumor cells, and endothelial cells (56). Notably, certain HMOs containing β1-3- or β1-4-linked galactose at their non-reducing end can potentially serve as targets for galectin-mediated interactions. A study employing catch-and-release electrospray ionization mass spectrometry (CaR-ESI-MS) examined the binding affinities of HMOs with three different human galectins Gal-1 (stable mutant of the protein), Gal-3 (C-terminal fragment of the protein), and Gal-7. The findings revealed that 25 of the 31 HMOs examined exhibit a high binding affinity to these galectins (57, 58). Another study has shown that oligosaccharides can also bind to Gal-2, Gal-8 and Gal-9 as well as the previously mentioned Gal-1, Gal-3 and Gal-7 (59). Carbohydrate Recognition Domains (CRDs) within Gal-9 were shown to have relatively high affinity for *N*-linked tri- and tetra-antennary complex-type glycans with LacNac units, only if these LacNac moieties were not fucosylated or sialylated (60). Prudden et al. furthered this by showing that HMOs with high affinity binding for Gal-9 had a LacNac extension on the β6- arm of the lactose core; predicting this is the point of binding between receptor and the HMOs (61). The possibility that HMOs have the capability to modulate galectin-mediated immune responses requires further investigation as these studies only highlight HMO affinity for galectins. Nonetheless, galectins represent promising candidates as potential receptors for HMO-mediated immune system modulation.

#### 3.1.2 Siglecs

Several studies have highlighted the potential interactions between HMOs and siglecs (Sialic-Acid-Binding Immunoglobulin-Like Lectins) which are known to bind sialylated glycans (62) and are considered regulators of the immune system (63). For example, certain HMOs containing sialic acid residues; such as 3’-SL and 6’-SL can serve as ligands for several siglecs expressed on immune cells (64). HMOs have been found to bind to half of the 14 siglecs currently identified as being expressed on human immune cells, albeit with relatively low affinity (64–68). By binding to siglecs, HMOs may modulate immune cell function, including activation, migration, and cytokine production. Furthermore, HMO-siglec interactions may influence immune tolerance (69) and inflammation in the gut. Siglecs expressed on immune cells can regulate immune responses to the gut microbiota and dietary antigens, and HMOs may play a role in this process by engaging with siglecs and modulating their signaling pathways. Similar to galectins, the downstream effects of HMO interactions with siglecs has yet to be elucidated.

#### 3.1.3 C-type lectin receptors

C-type lectin receptors (CLRs) are a diverse group of receptors expressed on various immune cells, including DCs, macrophages, and neutrophils. These receptors recognize specific carbohydrate structures, including those found on pathogens and host cells, and play critical roles in immune surveillance, pathogen recognition, and immune cell activation (70). Several studies have suggested that HMOs can interact with CLRs expressed on the surface of immune cells. HMOs may regulate immune cell activation, cytokine production, and antigen presentation by binding to these CLRs, thereby shaping the developing infant immune system. Furthermore, HMO-CLR interactions may also impact the gut microbiota composition and function, as CLRs that are expressed on intestinal epithelial cells and immune cells can recognize glycans of commensal bacteria and regulate their interactions with the host immune system. HMOs may influence these interactions by competing with bacterial glycans for CLR binding or by directly modulating CLR-mediated signaling pathways.

HMOs and their interaction with selectins, which are a subgroup of CLRs and are involved in various physiological and pathological processes, is an area of growing interest. Selectins are best known for their role in mediating the adhesion of immune cells to endothelial cells, facilitating their extravasation from the bloodstream into tissues during inflammation, and immune surveillance (71). Studies have demonstrated that certain fucosylated HMOs, are recognized by selectins expressed on the surface of immune cells (72). This suggests the involvement of HMOs in influencing selectin-mediated immune cell trafficking and function, reducing inflammation by blocking the trafficking of immune cells to site of infections (10).

Fucosylated HMOs can serve as ligands for CLRs such as Dendritic Cell-Specific Intercellular adhesion molecule-3-Grabbing Non-integrin (DC-SIGN) and Specific Intracellular adhesion molecule-Grabbing Non-integrin-Related (SIGN-R) expressed on DCs and macrophages. It has been shown that 2’-FL and 3-FL specifically were found to interact with DC-SIGN (68, 73). But again, downstream effects of these HMO interactions with CLRs are yet to be fully deciphered.

#### 3.1.4 Toll-like receptors

While the previously discussed immune receptors that have been shown to interact with HMOs are all glycan-specific lectin receptors, the interactions of HMOs with Toll-Like Receptors (TLRs) are different. TLRs are primarily distinguished as a class of Pathogen Recognition Receptors which recognize very specific Pathogen Associated Molecular Patterns (PAMPs).

Multiple studies have shown the interaction between HMOs and TLR4, a receptor known for recognizing bacterial lipopolysaccharides (LPS) (74). HMOs such as 3’-SL (75, 76) can competitively inhibit the activation of TLR4, thereby reducing inflammatory responses and promoting immune homeostasis in the infant gut. Whereas LNFP-III (77, 78) induces TLR4 activation leading to the maturation of type-2 DCs which induce a Th2-type T-cell response. This interaction helps prevent excessive immune activation and inflammation, contributing to the development of immune tolerance. In a study analyzing the effects of 2’-FL on β-lactoglobulin (β-LG) induced allergy, the study found that 2’-FL can directly inhibit the TLR4/NFκB pathway in a dose-dependent manner. In this way 2’-FL reduces the activity of TLR4, leading to decreased production of pro-inflammatory cytokines and a lower inflammatory response. Additionally, the study also highlighted that 2’-FL increases the expression of miR-146a, a microRNA known for its role in regulating immune responses. miR-146a can downregulate key proteins in the TLR4 signaling pathway, such as TNF receptor-associated factor-6 (TRAF6) and interleukin-1 receptor-associated kinase-1 (IRAK1) (79). By increasing miR-146a levels, 2’-FL further dampens the TLR4-mediated inflammatory response. Furthermore, when miR-146a inhibitors were added to macrophages, the inhibitory effect of 2’-FL on TRAF6 and IRAK1 expression in the TLR4 pathway was reduced, indicating that miR-146a plays a role in 2’-FL-dependent regulation of the immune response. These findings suggest that HMOs, like 2’-FL, may reduce β-lactoglobulin allergy by modulating miR-146a to inhibit the TLR4/NFκB signaling pathway (80) hindering the subsequent inflammatory response.

Other TLR interactions with HMOs have garnered less attention when compared with TLR4. HMOs, such as 3-FL and LNT2, have been shown to activate TLR2 (81) which is involved in recognizing lipid-containing microbial components (82), and the binding of HMOs to these receptors can modulate immune responses, potentially reducing inflammation and promoting immune tolerance. HMOs including LNT2 (81) and 3’-Galactosyllactose (3’-GL) (75) have also been found to interact with and inhibit TLR3, which recognizes viral double-stranded RNA (83). This interaction may contribute to the regulation of antiviral immune responses in the infant gut, helping to maintain immune homeostasis. LNT2, LNnT and 3-FL have been reported to also interact with TLR5 (81), which recognizes bacterial flagellin (84). HMOs may influence the immune response to flagellated bacteria in the gut by modulating TLR5 signaling, contributing to the maintenance of gut health. HMOs have been shown to interact with TLR9, which recognizes unmethylated CpG motifs present in bacterial DNA (85). This interaction may play a role in modulating immune responses to bacterial DNA in the infant gut, potentially influencing the development of immune tolerance. While the interaction of HMOs with TLR4 is well studied and the mechanism is relatively well understood, there is still further research to be conducted on the other TLRs and their mechanistic interactions with HMOs. These receptor-ligand interactions lay the groundwork for the downstream effects observed in immune cells, such as macrophages and dendritic cells, which are further explored in the next section. The specificity and affinity of HMOs for these receptors can explain their distinct immunomodulatory effects, serving as a mechanistic basis for their role in immune regulation.

### 3.2 Modulatory effects of human milk oligosaccharides on immune cells

HMOs are now being recognized to play a direct role in modulating immune response, acting as both direct and indirect defenders against pathogenic threats. The various types of immune cells known to interact with HMOs originate from both the innate (**Table 2**) and adaptive (**Table 3**) immune system, including macrophages, DCs, B-Cells, T-Cells, and NK cells.

**Table 2:** Effects of HMOs on cells of the innate immune system.

Cell Type	HMO	Effect	Ref
Macrophage	2’-FL	Increased expression of M2-genes and decreased expression of M1-genes	(87)
	LNFP-III	Activated macrophages and upregulated Gr1^+^ F4/80^+^ cells	(88)
	LNnT	Expanded anti-inflammatory macrophage cell type (Gr1^+^/CD11b^+^/F4/80^+^)	(89)
	Neutral HMOs	Decreased NO and PGE2 production, but stimulated ROS production	(90)
	Acidic HMOs	Increased ROS production but allowed for rapid return to basal level post infection	(91)
	2’-FL	Increased percentage of macrophage phagocytosis	(92)
	Acidic HMOs	Enhanced phagocytic activity	(91)
	6’-SL	Slightly increased phagocytic activity	(93)
	3’-SL 6’-SL	Reduced immune cell infiltration caused by LPS in the lung of ALI model	(94)
	6’-SL	Prevented the increase of macrophages in the cochlear spiral ganglion during neomycin otoxicity study	(93)
	HMO Mix	Increased monocyte and macrophage population in the MLN, lowered population in the spleen	(95)
	2’-FL LNFP-I	Decreased MNC activation by decreasing their proliferation	(96)
	3’-SL 6’-SL	Promoted production IL-10 and IL-4 while suppressing IL-1b, IL-8, TNF-α and IL-6	(94)
	2’-FL LNFP-I	Inhibited IFN-y and IL-12 and promoted IL-10	(96)
	LNnT	Promoted IL-10 and TGF-b production	(89)
	3-FL 3’-SL	Increased IL-6 secretion	(97)
	2’-FL	Increased levels of TNF-a	(97)
	LNT2	Increased levels of IL-12p70	(97)
Dendritic Cell	HMO Mix	Induced a partially mature state in human moDCs	(54)
	HMO Mix	Increased levels of IL-10, IL-27, and IL-6	(54)
	HMO Mix	Limited the maturation of moDCs induced by LPS and reduced production of IL-12p70, IL-6, and TNF-α	(54)
	2’-FL LNnT 6’-SL 3’-SL Mix	Less macrophages, pDCs, and mature DCs but more immature DCs	(98)
	3’-SL 6’-SL	increased the release of IL-12p70, IL-23, TNF-α, IL-6 and IL-10	(99)
	2’-FL	Increased production of IL-6	(100)
	3-FL 3’-SL 6’-SL	Increased the secretion of IL-6, IL-12p70, IL-23 and TNF-a	(97)
	3’-SL 6’-SL	Increased production of IL-10	(97)
	3-FL	Increased secretion of IL-6 and IL-23	(101)
	2’-FL	Increased activation of DCs in the spleen, Increased CD86^+^ and CD40^+^ expression	(103)
	2’-FL	Increased percentage of CD11c^+^ MHCII^+^ DCs	(103)
	2’-FL	Increased tolerogenic DCs (CD103^+^ CD11c^+^ MHC-II^+^) in MLN, as well as increased CD40^+^ expression and decreased CD86^+^ expression	(103)
	2’-FL LNnT 6’-SL 3’-SL Mix	Increased expression of co-stimulatory molecules but not MHC	(98)
Natural Killer Cell	2’-FL	Stimulated NK cell activity	(108)
	2’-FL	Increased IFN-y production	(108)
	2’-FL LNnT 6’-SL 3’-SL Mix	Increased PBMC NK cell population 2-fold	(98)
	LNFP-III	Activated NK cells by HMO-stimulated macrophages	(88)
Neutrophil	2’-FL	Decreased MIP2 production	(109)
	2’-FL	Reduced population of MPO7+ neutrophils	(109)
	Sialylated HMOs	Inhibited the adhesion and migration of neutrophils by binding to selectins	(10)
	HMO Mix	Increased neutrophil population in MLN short term, decreased this population longer term	(95)
	HMO Mix	Increased neutrophil population in the spleen	(95)
	2’-FL GOS	Enhanced the phagocytic capacity of neutrophils, improving internalization of *S. aureus* and *P. aeruginosa*.	(110)
	Sialylated HMOs	Decreased the oxidative burst of *H. pylori*-activated neutrophils	(111)
	LDFT	Suppressed the formation of platelet-neutrophil conjugates and the expression of neutrophil β2 integrin	(112) (113)
Mast Cell	2’-FL 3’-SL 6’-SL	Attenuated mast cell degranulation and release of histamine	( (80, 101, 114) ( (115, 116)
Basophil	2’-FL LNnT 6’-SL 3’-SL Mix	Increased PBMC basophil population 5-fold	(98)
Eosinophil	Acidic HMOs	Decreased lung eosinophilia upon challenge in FI-RSV-vaccinated mice	(119)

**Table 3:** Effects of HMOs on cells of the adaptive immune system.

Cell Type	HMO	Effect	Ref
T-Cell	HMO Mix	Decreased number of Tbet+ Th1 cells and IFN-γ production	(54)
	2’-FL	Induced IFNγ and IL-10 secretion by CD4^+^ T-cells	(102)
	2’-FL	Induced higher population of antigen specific CD4^+^ T-Cells in vaccine model	(103)
	Sialylated HMOs	increased IFN-γ producing CD4^+^ and CD8^+^ T-cells after long term exposure, as well as promote IL-13 producing CD8^+^ T-cells	(55)
	Acidic HMOs	Balanced Th1 and Th2 T-cell responses	(120)
	Sialylated HMOs	Increased a regulatory subpopulation of T-cells (CD25^+^/CD4^+^)	(55)
	HMO Mix	decreased CD4^+^ and CD8^+^ T-Cells short term but increased CD4^+^ population longer term	(95)
	HMO Mix	Increased the ratio of CD4^+^ to CD8^+^ T-Cells in MLN	(95)
	2’-FL LNnT 6’-SL 3’-SL Mix	Induced 36% more MLN effector memory T-cells	(98)
	2’-FL	Increased CD62L^+^ Th and CTL populations	(122)
	Sialylated HMOs	Decreased Th cell populations derived from PBMCs stimulated with LPS	(123)
	2’-FL LNnT BMOs	Modulated Th cell population	(124)
	2’-FL	Reduced the proportion of Th17 cells and suppressed Th17-related cytokine production	(125)
	2’-FL	Inhibited the differentiation of Th17 cells, STAT3 phosphorylation, and RORγt mRNA levels	(125)
	Pooled HMOs	Induced Tregs and increased IL-10 production, reduced Th1 frequency and IFN-γ production	(54)
	Sialylated HMOs	Enhanced the secretion of IFN-γ and IL-17A from CD4^+^ T-Cells, reduced IL-13 production	(97)
	LNFP-III LNnT	Repressed Th1 cell proliferation; by promoting the expansion of Gr1+ macrophages	(89)
	3’-SL	Increased proportion of Tregs and upregulated RORyT expression	(126)
	2’-FL	Reduced Th17-related cytokine production	(125)
	2’-FL	Induced 13% higher CD4^+^ and 7.4% higher CD8^+^ T-cells in vaccine model	(128)
	3’-GL	Increased Th1 cell activation and attenuated deoxynivalenol (DON)-induced effects in Th1 cell responses	(129)
	2’-FL scGOS lcFOS	Increased influenza vaccine specific DTH responses, Tregs, and Th1 cells in their MLNs	(130)
	Acidic HMOs	Reduced RSV-specific Th2 cytokine-producing CD4^+^ T-Cells short term and increased IFN-γ-producing CD4^+^ and CD8^+^ T-Cells longer term	(119)
B-Cell	2’-FL	Increased activated B-cells (CD19^+^ B220^+^) in both the spleen and MLNs	(103)
	2’-FL	Increase in the percentage of activated B-cells expressing CD27 in the spleen	(103)
	HMO Mix	Increase plasma cells (B220- CD138^+^) in MLNs	(95)
	HMO Mix	Increased B-cell numbers and the plasma cell population was higher	(95)
	HMO Mix	Increased antibody-secreting cells for TT and DT antigens in the spleen	(95)
	2’-FL	Increased IgG2a and IgG1 antibody levels	(103)
	2’-FL scGOS lcFOS	Increased IgG2a production	(131)
	2’-FL	Heightened plasma IgG levels, of IgG2a, IgG2b and IgG2c	(123)
	2’-FL	Induced 40% rise in plasma IgA levels	(123)
	2’-FL	Increased caecal sIgA levels and lower total serum IgE levels	(133)
	2’-FL	Elevated serum total IgG levels	(133)
	2’-FL scGOS lcFOS	Increased trivalent influenza vaccine (TIV)-specific IgG and IgG1	(134)

#### 3.2.1 Cells of the innate immune system

##### 3.2.1.1 Macrophages

Macrophages are phagocytic cells derived from monocytes that play a crucial role in both innate and adaptive immunity. They engulf and digest pathogens, debris, and dead cells, acting as scavengers and antigen-presenting cells (APCs). Macrophages also secrete cytokines and chemokines to regulate immune responses, promote inflammation, and coordinate tissue repair (86). HMOs can influence the activation state of macrophages and promote the polarisation of macrophages towards an anti-inflammatory M2 phenotype. Data from a study analysing macrophages in an ischemic stroke mouse model indicated that 2-’FL increases the expression of M2-type genes and decreased M1-type genes (87). LNFPIII activates macrophages *in vitro* as indicated by upregulation of Gr-1 expression in F4/80+ cells (88). Additionally, macrophages exposed to LNnT expanded a cell population, phenotypically defined as Gr1^+^/CD11b^+^/F4/80^+^ which typically possess an anti-inflammatory cytokine profile (89). Using the murine macrophage-like RAW264.7 cell line, a specific fraction of neutral HMOs isolated from breast milk decreased production of nitric oxide (NO) and Prostaglandin-E2 (PGE2) (53) which have been shown to stimulate macrophage polarisation towards a M2 phenotype (90).

In this same study the isolated HMO fraction was observed to stimulate the release of reactive oxygen species (ROS) in RAW264.7 macrophages which aids in bacterial clearance. The study also demonstrated that this fraction activates macrophages through the NFκB and MAPK signalling pathways. Further studies analysing *Pseudomonas aeruginosa* K (PAK) infection in THP-1 macrophage-like cells, showed an increase in ROS production but additionally displayed the rapid return of ROS levels to basal amounts post clearance of infection (91). These results show that HMOs elicit the ROS production to aid in bacterial clearance by macrophages but do not allow for enhanced inflammation due to prolonged ROS production.

HMOs have also been found to enhance the phagocytic activity of macrophages, increasing their ability to engulf and eliminate pathogens. In one study, 2′FL intervention resulted in the higher percentage of macrophage phagocytosis in offspring of supplemented mice as compared to the control group (92). This enhanced phagocytosis may contribute to the overall antimicrobial activity of macrophages and aid with more effective infection clearance. Another study also showed that sialylated HMOs promoted bacterial clearance through enhanced phagocytic activity (91) thought to be elicited by the presence of sialic acid binding receptors present on macrophages promoting phagocytosis. However, a different study investigating THP-1 cells treated with 6’-SL showed that 6’-SL alone slightly increased phagocytic activity. Cells treated with LPS and 6’-SL, actually decreased phagocytosis compared to cells treated with LPS alone and subsequently a decrease in ROS production was observed with LPS and 6’-SL treated cells (93).

As well as altering physical attributes of macrophage function, HMOs have also been shown to inhibit the infiltration of macrophages to sites of inflammation and also alter population distribution. A study analysing a model of acute lung injury (ALI) demonstrated that mice that were intraperitoneally administered with 3’-SL and 6’-SL prior to LPS challenge had significantly reduced immune cell infiltration caused by LPS (94). Additionally, a study analysing the ototoxic effect of neomycin demonstrated that 6’-SL prevented the increase in number of macrophages in the cochlear spiral ganglion (93). This HMO driven mechanism in reducing the infiltration of macrophages to the site of injury or infection, may help limit tissue damage and promote resolution of inflammation. HMOs may also induce changes in population distribution of monocytes and macrophages as these cell populations were observed to be higher in the mesenteric lymph nodes (MLN) with 7-day HMO supplementation of C57BI/6 orally gavaged with 15 mg/day in an *in-vivo* study, while in the spleen, this population was lower in all HMO-supplemented groups compared to their control (95). 2’-FL and LNFP-I, used in a concentration which could be considered physiological, negatively influenced mononuclear cells (MNC) activation by decreasing their proliferation in a dose-dependent fashion (96).

HMOs can also modulate inflammatory responses in macrophages by influencing the production of inflammatory mediators. Some studies propose that HMOs may modulate cytokine production in immune cells towards a more balanced and less inflammatory state. For instance, certain HMOs have been shown to promote the production of anti-inflammatory cytokines such as IL-10 and IL-4 while suppressing the secretion of pro-inflammatory cytokines such as IL1b, IL8, TNF-α and pleiotropic IL-6 (94). 2’-FL and LNFP-I have an inhibitory role on IFN-γ and IL-12 production *ex vivo* particularly in MS patients, and prime release of the Th2 cytokine IL-10, displaying an immunomodulatory effect *ex vivo* (96). A LNnT treated Gr1+/F4/80+ population of macrophages produced lower levels of proinflammatory cytokines, but higher levels of IL-10 and TGF-β *ex vivo*, compared to the untreated control (89). These findings imply that HMOs could contribute to immune homeostasis and mitigate excessive inflammatory responses helping regulate immune responses. However, other studies in contrast, suggest that the effects of HMOs on cytokine production are inconsistent or even pro-inflammatory in certain cases. For example, in a study analysing LPS activated monocyte-derived macrophages, 3-FL and 3′-SL increased IL-6 secretion. High doses of 2′-FL increase the levels of TNF-a and LNT2 increasing levels of IL-12p70. These results demonstrate that sialylated HMOs and 3-FL added to classically-activated M1 macrophages enhance pro- and anti-inflammatory cytokine production (97).

Specific HMO structures may enhance the production of pro-inflammatory cytokines under certain conditions, potentially exacerbating inflammatory responses. It seems generally that fucosylated HMOs influence an anti-inflammatory cytokine profile, whereas sialylated HMOs have more of a pro-inflammatory cytokine output. The effects of HMOs on cytokine production may vary depending on factors such as cell type, concentration, and the presence of other immune-modulating molecules. These contextual factors can influence the direction and magnitude of immune responses to HMOs and therefore may warrant further research to elucidate the specific mechanisms of action.

##### 3.2.1.2 Dendritic cells

Dendritic cells (DCs) are professional APCs that play a pivotal role in initiating adaptive immune responses. They capture antigens in peripheral tissues, process them, and migrate to lymphoid organs where they present antigens to T-Cells, in turn activating them. DCs bridge the innate and adaptive immune systems by linking recognition of pathogens to the activation of specific immune responses (86). HMOs can influence the maturation state of DCs. A mixture of HMOs derived from human milk samples were found to induce a partially mature state in human monocytes-derived DCs (moDCs), resulting in increased levels of anti-inflammatory IL-10, IL-27, and IL-6, but not pro-inflammatory IL-12p70 and TNF-α (54). These HMO-treated moDCs, in turn, facilitate the generation of regulatory T-Cells (Tregs) from naïve CD4^+^ T-cells. Notably, this study found that HMOs limited the maturation of human moDCs induced by LPS while maintaining secretion of IL-10 and IL-27 and reducing production of IL-12p70, IL-6, and TNF-α triggered by LPS. This also resulted in higher number of Tregs, while showing a decrease in the number of Tbet^+^ Th1 cells and IFN-γ production compared to LPS-stimulated moDCs (54). In an *in vivo* study, peripheral blood mononuclear cells (PBMCs) and MLNs of HMO-fed non-infected pigs had three times less plasmacytoid DCs (pDCs) than those of formula-fed non-infected pigs. In the MLNs, the HMO-fed non-infected pigs had less macrophages, pDCs, and mature DCs but more immature DCs than formula-fed non-infected pigs (98) indicating a potential role of HMOs in modulating DC populations and immune responses.

HMOs can also influence the cytokine production profile of DCs. In one such study, 3′-SL and 6′-SL increased the production of key pro-inflammatory cytokines IL-12p70, IL-23 and TNF-α, and anti-inflammatory IL-6 (in this case) and IL-10 from LPS-activated DCs. 2’-FL has also been shown to induce IL-6 production in DCs (99, 100). In LPS-activated moDCs, 3-FL, 3’-SL, and 6’-SL led to a marked increase in the secretion of IL-6, IL-12p70, IL-23, and TNF-α. However, only the sialylated HMOs caused a significant rise in IL-10 secretion (97). 3-FL showed a modest immunomodulatory effect by significantly boosting IL-6 and IL-23 secretion levels (101). These findings, along with other studies, highlight that both the sialylated and fucosylated HMOs enhance production of pro- and anti-inflammatory cytokines in activated DCs, in turn influencing T-cell polarization thus promoting Th1, Th17, and regulatory immune responses (76, 97).This differs from the previous study as individual sialylated HMOs are investigated rather than a mixture of unknown HMOs in unknown quantities isolated from breast milk. MoDCs exposed to 2′-FL and CpG-conditioned Intestinal Epithelial Cells (IECs) induced IFNγ and IL-10 secretion by CD4^+^ T-cells, promoting the development of a Treg response (102). This modulation of cytokine production by DCs may contribute to the overall regulation of immune responses by HMOs.

HMOs have been reported to promote immune tolerance in DCs. By inducing the production of anti-inflammatory cytokines and inhibiting the release of pro-inflammatory mediators, HMOs may help dampen DC-mediated immune responses and promote the development of immune tolerance (54). In mice fed with 1% 2′-FL increased activation of DCs was present in the spleen, evident by higher levels of CD86^+^ and CD40^+^ markers. There was also a slight increase in the percentage of CD11c^+^ MHCII^+^ DCs. In the MLNs, 2′-FL led to an increase in tolerogenic DCs (CD103^+^ CD11c^+^ MHC-II^+^), known for inducing Tregs and gut-homing molecules. Additionally, CD86 expression decreased while CD40 expression increased in MLN DCs, suggesting a shift towards a more tolerogenic phenotype (103). Another study showed that HMOs altered the expression levels of programmed-death-ligand-1 (PD-L1) and OX40-L both of which are expressed on healthy APCs such as DCs (104, 105) and were significantly increased in the presence of LPS and HMOs as compared to untreated immature DCs, pointing to the potential of HMOs inducing Tregs and skewing immune responses (106) towards homeostasis.

HMOs can affect the antigen-presenting function of DCs and modulate the expression of co-stimulatory molecules (107) but not major histocompatibility complex (MHC) (98) molecules on DCs. This was further confirmed by demonstrating in a vaccine model, that 2’-FL induced higher population of antigen specific CD4^+^ T-Cells compared to the untreated control vaccine model (103). This regulation of antigen presentation of DCs by HMOs may influence the activation and differentiation of T-Cells and shape the overall immune response.

##### 3.2.1.3 Natural killer cells

Natural killer (NK) cells are a type of cytotoxic lymphocyte that plays a critical role in innate immunity and defence against viruses and tumor cells. NK cells can directly recognise and kill virally infected or abnormal cells without prior sensitisation. They also secrete cytokines that help regulate immune responses and stimulate other immune cells (86). HMOs have been found to enhance the activity of NK cells, particularly their cytotoxicity against infected or abnormal cells. Studies have shown that certain HMOs, such as 2’-FL, can stimulate NK cell activity (108) potentially increasing their ability to recognise and kill target cells. Additionally, HMOs can influence the phenotype of NK cells, affecting their activation status and cytokine production profile such as IFN-γ (108). This modulation of NK cell phenotype may enhance their effector functions and contribute to the regulation of immune responses. As well as altering the phenotype of NK cells HMOs may regulate the activation and differentiation of NK cells. In an *in-vivo* study HMO-fed pigs had 2-fold increase in PBMC NK cells (98). This regulation of NK cell activation and differentiation may influence their functional properties and contribute to the overall immune response. Not many studies have analysed the direct effect of HMOs on NK-cells specifically, but a study has shown that the activation of NK cells by HMO-stimulated macrophages, specifically by LNFP-III, requires cell-cell contact. The authors concluded that NK cell activation is macrophage-dependent, as NK cells alone did not respond to LNFP-III (88).

Very little research has been conducted analysing the effect of HMOs on NK cells. This may pose an opportunity for future research as NK cells are pivotal in innate immunity and greatly aid in the clearance of infection through Antibody-Dependent Cellular Cytotoxicity (ADCC), a process by which NK cells recognise and kill antibody-coated target cells through the production of granzyme and perforin. One avenue of investigation could focus on elucidating how HMOs modulate Fc-receptor signalling on NK cells as HMOs may influence the affinity or avidity of Fc-receptors for antibody-bound targets, thereby impacting the efficacy of NK cell-mediated ADCC. Additionally, HMOs might directly affect the activation status or cytotoxic potential of NK cells, altering their ability to recognize and eliminate antibody-coated cells. By elucidating these interactions, we could gain deeper insights into the multifaceted benefits of breastfeeding and improve innovative approaches to enhance immune defence in cases of viral infection.

##### 3.2.1.4 Neutrophils

Neutrophils are the most abundant type of white blood cell and are essential for innate immunity. These cells engulf and destroy pathogens, particularly bacteria and fungi, through phagocytosis. Neutrophils also release antimicrobial proteins and ROS to kill pathogens and modulate immune responses (86). Research has indicated that HMOs influence the chemotactic behaviour of neutrophils, dampening their capacity to migrate towards areas of infection or inflammation. Specifically, studies have illustrated that 2’-FL, diminished the expression of chemokines, such as macrophage-inflammatory-protein-2 (MIP-2), which are crucial for neutrophil recruitment. Furthermore, investigations have revealed a reduction in the number of myeloperoxidase-7 (MPO7)+ neutrophils when treated with 2’-FL, impeding their recruitment to sites of tissue damage or microbial invasion (109), thereby controlling excessive inflammation at sites of infection. Furthering the effect of HMOs on neutrophil migration, HMOs have been found to inhibit the adhesion and migration of neutrophils by binding to selectins (10), which as mentioned are cell adhesion molecules involved in the recruitment of neutrophils to sites of inflammation. HMOs do this by specifically interfering with the interaction between selectins on endothelial cells and their ligands on neutrophils. This reduces the ability of neutrophils to adhere to and migrate across the endothelium, subverting innate immune responses. HMOs potentially mitigate tissue damage and inflammation by attenuating excessive neutrophil infiltration into inflamed tissues associated with conditions such as infection, injury, or autoimmune diseases.

As well as preventing their recruitment to sites of inflammation, HMOs have also been shown to alter the population of neutrophils in certain immune organs. A study analysing the effects of HMOs feeding in 3-week-old C57BL/6 germ-free (GF) mice found that in the mesenteric lymph nodes (MLN), neutrophil numbers were higher in the group fed HMOs for 7 days, measured at 28 days after birth. However, by day 50 post-birth, the group that received 14 days of HMO feeding showed a lower neutrophil population compared to the control group (95). The neutrophil population in the spleen was decreased by both 7- and 14-day HMO supplementation at day 28, 35 and 50 post birth. These results indicate that HMOs can influence the distribution of lymphocyte subpopulations, even in GF mice which notably lack a microbiota, and that these effects may also be tissue specific.

HMOs can also modulate the phagocytic activity of neutrophils, affecting their ability to engulf and eliminate pathogens. HMOs enhance the phagocytic capacity of neutrophils, improving their ability to recognise and internalise microbial pathogens (110), such as *S. aureus* and *P. aeruginosa*. Furthermore, HMOs modulate the generation of ROS by neutrophils, influencing cellular signaling pathways and the oxidative burst associated with immune responses. Sialylated oligosaccharides decrease the oxidative burst of *H. pylori*-activated neutrophils (111). This suggests a potential strategy to disrupt various stages of *H. pylori* infection. The intricate mechanisms of this infection involves neutrophil activation leading to oxidative bursts, which induces tissue damage during infection. Therefore, inhibiting neutrophil activation and subsequently oxidative bursts may be advantageous in preventing these damaging effects.

HMOs have demonstrated the ability to suppress microvascular inflammation in neutrophils, particularly associated with the formation of platelet-neutrophil conjugates and the expression of neutrophil β2 integrin (112). This suggests that HMOs could mediate suppression of platelet pro-inflammatory functions via neutrophil action (113).

It may be worth studying the effects of HMOs on the production of Neutrophil extracellular traps (NETs) which are web-like structures composed of DNA, histones, and antimicrobial proteins released by neutrophils to trap and kill pathogens. Excessive NET formation has been associated with tissue damage and inflammation and therefore would be worth noting whether HMOs influence their production, potentially limiting tissue damage and inflammation associated with excessive neutrophil activation.

##### 3.2.1.5 Other innate immune cells

Basophils, mast cells and eosinophils are granulocytes involved in allergic reactions and inflammatory responses. Basophils and mast cells release histamine and other mediators in response to allergens, pathogens, or tissue damage, leading to vasodilation, increased vascular permeability, and recruitment of other immune cells to the site of inflammation (86). Eosinophils are involved in immune responses against parasitic infections and allergic reactions. They release cytotoxic granules containing enzymes and proteins that kill parasites and modulate allergic inflammation. Eosinophils also participate in tissue repair and immune regulation (86).

HMOs can modulate the activation of mast cells, which play a central role in allergic responses and the initiation of inflammation. Studies suggest that certain HMOs; 2’-FL, 3-FL and 6’-SL attenuate mast cell degranulation (80, 101, 114) and the release of inflammatory mediators, such as histamine, prostaglandins, and cytokines (115, 116). By inhibiting mast cell activation and the release of allergic mediators, HMOs may help reduce the severity of allergic symptoms and inflammatory responses associated with conditions such as allergic rhinitis, asthma, and atopic dermatitis.

HMOs have not directly been shown to inhibit the activation of basophils, which are involved in allergic reactions and the release of histamine and other inflammatory mediators. An *in-vivo* study showed that HMO-fed pigs had five times more PBMC basophils than formula-fed pigs (98). It should be noted that butyrate has been shown to decrease basophil action (117), and HMO metabolism by certain beneficial bacteria in the gut generates increased (118) SCFAs and butyrate synthesis. Certain HMOs may interfere with the signalling pathways that lead to basophil activation, thereby attenuating allergic responses and hypersensitivity reactions. HMOs may also reduce the release of histamine from activated basophils, which can help alleviate symptoms of allergic reactions, such as itching, swelling, and bronchoconstriction.

Currently, there is a lack of research demonstrating the influence of HMOs on the activation of eosinophils, which are crucial in allergic responses and defence against parasitic infections. Dietary intervention however, with acidic HMOs decreases lung eosinophilia upon challenge in FI-RSV-vaccinated mice (119). It is plausible that HMOs could mitigate eosinophil activation, thereby diminishing their release of inflammatory mediators such as histamine and leukotrienes. Further, HMOs might aid in attenuating eosinophil-driven inflammation by curbing the production of pro-inflammatory cytokines and chemokines. Through such modulation of eosinophil function, HMOs could play a role in regulating allergic responses and resolving inflammation. Hence, exploring the specific interaction between these oligosaccharides and this immune cell subset warrants further investigation.

#### 3.2.2 Cells of the adaptive immune system

##### 3.2.2.1 T-cells

T-Cells are a type of white blood cell that play a central role in cell-mediated immunity. They can be further divided into several subsets, including CD8^+^ cytotoxic T-Cells (CTLs), CD4^+^ helper T-Cells (Th1, Th2, Th17, Tfh), and regulatory T-Cells (Tregs). CTLs destroy infected or abnormal cells, Th cells assist other immune cells by releasing cytokines and activating B-Cells, while Tregs help maintain immune tolerance and prevent autoimmune reactions (86). The interaction between HMOs and T-Cells is an intricate one. With numerous HMO variants potentially engaging with these circulating lymphocytes, HMOs are known to regulate the balance between promoting pro- and anti-inflammatory T-Cell responses. Different HMOs may induce varied alterations in T-Cell phenotypes, and these effects can be context-specific, depending on whether the immune system is in a state of homeostasis or facing an infection. Taking this into consideration, studies involving HMOs interaction with T-Cells have led to varied results.

HMOs have the ability to modulate the immune system and reduce inflammation, as they may skew T-cells to balance Th1 and Th2 responses (120). Sialylated HMOs derived from pooled human milk were shown to increase IFNγ producing CD4^+^ and CD8^+^ T-cells after long term exposure, as well as promote IL-13 producing CD8^+^ T-cells derived from human cord blood MNC (55). This study also revealed that these acidic HMOs increased the amount of CD25^+^/CD4^+^ T-cells, which are a regulatory subpopulation of T-cells. This was the first indication that HMOs encouraged the establishment of a Th1/Th2 balance. These findings suggest that HMOs impact cytokine production and activation of cord blood-derived T-Cells *in vitro*. Hence, oligosaccharides, particularly acidic structures, may influence lymphocyte maturation in breastfed newborns. An *in-vivo* study demonstrated that 7 and 14 days of HMO supplementation led to a reduction in CD4^+^ and CD8^+^ T-cells in the MLN at 28 and 35 days when compared to GF controls. However, by day 50, the CD4^+^ T-cell population had increased in both the 7- and 14-day HMO-treated groups in the MLN. In the spleen, no significant differences were noted at days 28 or 35, but CD4^+^ T-cell numbers rose at day 50 in both HMO-treated groups. Additionally, the CD4^+^ to CD8^+^ T-cell ratio in the MLN increased at day 35 following 14 days of HMO treatment (95). This agrees with another *in-vivo* study where HMO-fed pigs were found to have 36% more MLN effector memory T-Cells compared to the control feed group (98). Memory T-Cells are antigen-experienced cells that mediate a faster and more potent response upon repeat encounter with antigen (121) and therefore HMOs may increase this population to aid in the rapid clearance of certain recurring infection in infants.

HMOs can influence the differentiation of T-Cells into different effector subsets. Neonatal rats given daily doses of 2’-FL showed an elevated proportion of T-cells, in both Th and CTL populations. The 2’-FL treatment resulted in a higher percentage of CD62L^+^ Th lymphocytes. CD62L also known as L-selectin is a key cell adhesion molecule on lymphocytes and facilitates their migration to lymph nodes for immune surveillance, playing an essential role in initiating immune responses by allowing T-cells to exit the bloodstream (122). 2’-FL did not however, affect T-cells expressing the activation marker CD25. Additionally, the CTL increase was seen in both TCRαβ+ and TCRγδ+ subsets (123). *Ex vivo* HMO co-stimulation affected Th cell populations in PBMCs and decreased Th cell populations in response to LPS stimulation. Co-stimulation with isolated HMOs decreased Th cell populations derived from PBMCs stimulated with LPS. This study found that co-stimulation with a sialyllactose mix, or isolated HMO decreased CD4^+^CD8^+^ T-cell populations under conditions that would normally stimulate T-Cells (124). Interestingly, a different study analysing HMOs and bovine milk oligosaccharides (BMOs) found that feeding 2’-FL and LNnT or sialic acid-enriched BMOs to 2-day-old pigs through supplemented diets did not alter the profile of PBMC lymphocyte populations. The study findings indicated a synergistic effect of both BMOS and HMOs on Th cell populations (125) suggesting that a more complex combination of oligosaccharides, similar to what is found in human milk, is needed to produce a Th phenotypic cell distribution in formula-fed animals similar to that of breastfed infants. Treatment of female 6-week-old C57BL/6J mice with 2′-FL reduced the proportion of Th17 cells and suppressed Th17-related cytokine production in a skin-inflammation mouse model. Additionally, 2′-FL inhibited the phosphorylation of STAT3 in skin tissue, reducing recruitment of Th17 cells. *In vitro* work in this same study culturing naïve CD4^+^ T-cells found that 2′-FL inhibited the differentiation of Th17 cells, STAT3 phosphorylation, and RORγt mRNA levels in T-Cells under Th17 polarization (126).

HMOs have been shown to modulate T-Cell activation by directly interacting with T-Cells or by affecting the function of APCs such as DCs. Certain HMOs, such as 2’-FL, have been found to enhance T-Cell activation by promoting the maturation and antigen-presenting capacity of DCs, which subsequently leads to an increase in T-Cell proliferation and cytokine production. HMO-conditioned human moDCs promoted Treg generation from naive CD4^+^ T-Cells. Furthermore, HMOs+LPS stimulated DCs induced a higher frequency of Tregs and increased IL-10 production, while a reduction in Th1 frequency and IFN-γ production was detected when compared to LPS-DCs (54). Studies have also suggested that acidic HMOs enhanced the secretion of IFN-γ and IL-17A from CD4^+^ T-Cells primed by activated DCs and macrophages while reducing the secretion of IL-13. Thus, these sialylated HMOs 3′-SL and 6′-SL support Th1 and Th17 responses while reducing Th2 responses (97). HMOs may induce Th1 and Th17 T-Cell phenotypes over Th2 to support host defence, tissue repair, and balanced immune responses in breastfed infants. In the MLNs, 2′-FL led to an increase in tolerogenic DCs, which subsequently induce Tregs and gut-homing molecules (103). However certain HMOs, such as LNFP-III and LNnT have also been observed to indirectly repress Th1 cell proliferation; doing so by promoting the expansion of Gr1+ cells which suppresses naive CD4^+^ T-Cell proliferation *ex vivo*. Coculture with Gr1^+^ cells does not induce CD4^+^ T-Cell apoptosis but leads to reduced IFN-γ levels and increased IL-13 production (89). These findings suggest a ligand-specific mechanism of HMOs, resulting in the generation of anti-inflammatory mediators that suppress Th1-type and inflammatory responses.

HMOs have also been reported to induce T-Cell tolerance, which is important for preventing autoimmune reactions and maintaining immune homeostasis. By promoting the differentiation and activation of Tregs, HMOs can help suppress excessive immune responses and prevent autoimmune diseases. Offspring of mothers with gestational diabetes mellitus (GDM) were treated with 3′-SL resulting in a significant increase in the proportion of Treg cells and upregulation of RORγt expression (127). RORγt is crucial for maintaining mucosal tolerance in the gut, and RORγt^+^ Tregs play a direct role in protecting against food allergy (128). This indicates that administering HMOs to GDM offspring promotes the development of immune tolerance in newborns.

HMOs can modulate the production of cytokines by T-Cells, influencing the balance between pro- and anti-inflammatory immune responses. Studies have shown that certain HMOs can enhance the production of anti-inflammatory cytokines such as IL-10 while reducing the production of pro-inflammatory cytokines such as IFN-γ and IL-17 by T-Cells. Treatment with 2′-FL reduced Th17-related cytokine production in a skin-inflammation mouse model. These findings suggest that 2′-FL alleviates psoriasis by modulating the Th17 immune response and cytokine secretion which the authors suggested to be via the STAT3 signalling pathway (126).

When discussing modulation of T-Cell function, it is important to consider implications for vaccine efficacy. T-Cells play a crucial role in vaccine-mediated immunity, particularly in generating long-term protection against pathogens. Therefore, the question arises as to whether HMOs may alter or promote vaccine function through T-Cells. Female 6-week-old C57BL/6JOlaHsd mice in a murine influenza vaccination model receiving high supplemented 2′-FL diet, had a 13% higher CD4^+^ and a 7.4% higher CD8^+^ T-cell population detected in comparison to the control vaccinated group, suggesting dietary intervention with 2′-FL increased vaccine-specific T-cell responses. Administration of acidic oligosaccharides boosted vaccine-specific delayed-type hypersensitivity (DTH) responses in a dose-dependent manner *in vivo* by measuring ear swelling in mice 24hrs post antigen injection. This coincided with a decrease in the production of Th2 cytokines by splenocytes *ex vivo* (129). The findings suggest that the dietary intervention shifted the systemic immune response to the vaccine towards a Th1-skewed profile. Further to this, another study demonstrated the dietary supplementation of the HMO 3’-GL increased Th1 cell activation and attenuated deoxynivalenol (DON)-induced effects in Th1 cell responses in a preclinical influenza vaccine model (130). Another dietary study in 6 week-old female C57BL/6JOlaHsd mice, containing 2’-FL with short-chain galactooligosaccharides (scGOS) and long-chain fructooligosaccharides (lcFOS) resulted in increased influenza vaccine specific DTH responses, Tregs, and Th1 cells in their MLNs (131). Additionally, in a Th2-skewed formalin-inactivated respiratory syncytial virus (FI-RSV) vaccination model in mice, acidic HMOs were shown to reduce RSV-specific Th2 cytokine-producing CD4^+^ T-Cells 4- and 6-days post-infection. This was accompanied by increased IFN-γ-producing CD4^+^ and CD8^+^ T-Cells at 8-days post-infection. These results indicate that particular dietary oligosaccharides may affect the trafficking and effector functions of CD4^+^ and CD8^+^ T-cell subsets in the lungs of mice infected with RSV (119). This is interesting as we enter into a post antibiotic era where a heightened response is needed to develop vaccines for multi-drug resistant (MDR) bacterial species, especially for ESKAPE pathogens which are a group of bacteria known for their ability to “escape” the effects of antibiotics. ESKAPE pathogens include *Enterococcus faecium, Staphylococcus aureus, Klebsiella pneumoniae, Acinetobacter baumannii, Pseudomonas aeruginosa* and *Enterobacter species*. Enhancing Th1 effector T-cell responses is proposed as a key target for vaccine development, given that this T-cell subset is crucial for effectively clearing infections. Memory Th1 cells provide protection against invasive *S. aureus* infection (132) which a vaccine is desperately needed against. Since HMOs have been shown to boost Th1 cell responses, incorporating these oligosaccharides into a vaccine could be a viable approach to developing a much-needed vaccine against critical pathogens, including *S. aureus.*

##### 3.2.2.2 B-cells

B-Cells are another type of white blood cell that are primarily responsible for humoral immunity. When activated by antigens, B-Cells differentiate into plasma cells, which produce antibodies. Antibodies bind to specific antigens, marking them for destruction by other immune cells or neutralising their effects directly. B-Cells also have memory cells that provide long-lasting immunity against previously encountered pathogens (86). HMOs have been reported to promote the survival and differentiation of B-Cells into antibody-secreting plasma cells. One study, providing dietary 2’-FL to 6-week-old female C57BL/6JOlaHsd mice 2 weeks prior to a subcutaneous vaccination found that supplementing with varying concentrations of 2′-FL induced higher frequencies of activated B-cells (CD19^+^ B220^+^) in both the spleen and MLNs compared to controls. Additionally, in the spleen, there was an increase in the percentage of activated B-cells expressing CD27 (103). In 3-week-old GF C57BL/6 mice orally gavaged with HMO supplementation had an increase in plasma cells (B220- CD138^+^) in MLNs at days 28, 35, and 50. In the spleen, B-cell numbers at day 50 increased and the plasma cell population was higher at days 28 and 50 with 7-day HMO supplementation. When assessing *ex vivo* MLN and spleen immune responses to specific antigens, Cholera Toxin B (CTB), Tetanus Toxin (TT), and Diphtheria Toxin (DT), no differences were observed between the GF and HMO-immunised groups for CTB in MLN. However, there were increased antibody-secreting cells (ASCs) for TT and DT antigens in the spleen of HMO-immunised mice compared to GF-immunised mice (95). By providing a supportive microenvironment for B-cell activation and proliferation, HMOs could enhance the generation of plasma cells and the production of antibodies, contributing to the humoral immune response.

HMOs can influence the production of antibodies by B-Cells. Studies have suggested that HMOs may enhance the production of specific types of antibodies, such as immunoglobulin A (IgA), which are important for mucosal immunity and defence against pathogens at mucosal surfaces (133). Additionally, HMOs may influence the specificity and affinity of antibodies produced by B-Cells, contributing to the diversity of the antibody repertoire. In mice, feeding with 2′-FL increased IgG2a and IgG1 antibody levels (103) and this was confirmed with other studies also showing increased IgG2a production in HMO supplemented diets (131). *In vivo*, HMO supplements have shown promising results for antibody modulation. For example, in one study animals receiving 2′-FL supplementation exhibited heightened plasma IgG levels, notably a 50% increase in IgG2b 8-days into supplementation. By day 16, this trend persisted with further elevations in IgG2a and IgG2c levels. Additionally, 2′-FL supplementations led to an over 40% rise in plasma IgA levels compared to the control group at day 16, although no significant impact on IgM was found (123). Separate studies showed that in BALB/c mice 21-days post-birth, 2′-FL supplementation resulted in significantly higher cecal sIgA levels and lower total serum IgE levels compared to the control group. By 56 days post-birth, the group treated with B79 + 2′-FL exhibited significantly elevated serum total IgG levels compared to the control group (134). Finally, an influenza vaccine mouse model study showed that at day 28 after primary vaccination a significant increase in trivalent influenza vaccine (TIV)-specific IgG and IgG1 was observed in offspring fed a scGOS/lcFOS/2’-FL diet from weaning compared to the control group. The group receiving scGOS/lcFOS/2’-FL from pregnancy also showed increased TIV-IgG1 levels. At day 49, scGOS/lcFOS/2’-FL treated mice from birth or weaning displayed an increase in TIV-specific IgG1. Interestingly, these effects were significant in male offspring but not in female mice (135). Moreover, HMO supplementation led to higher antigen specific antibodies as shown by higher serum DT-IgG levels compared to GF-immunised mice (95).

These studies have revealed a notable trend towards heightened levels of IgA and IgG immunoglobulins, which are recognized for their beneficial roles in combating infection and facilitating its clearance, both in the gut and also systemically. Concurrently, there appears to be a discernible decrease in IgE levels following HMO treatment, which is closely associated with allergic responses. This suggests that there is an intricate interplay between HMOs and their influence on specific antibody production, that may be context specific. There is no evidence to show HMOs modulate the activation of B-Cells by directly interacting with B-Cell surface receptors. This may warrant further investigation and may elucidate how HMOs induce the production and secretion of immunoglobulins for the protection of the infant.

### 3.3 Indirect implications of human milk oligosaccharides on immune system function

Beyond their direct effects on immune cells, HMOs influence broader immune functions indirectly. A significant mechanism is via the gut microbiota whereby HMOs intricately shape the gut microbiota composition, fostering a beneficial bacterial repertoire that influences various immune pathways. Whether by promoting health-associated microbiota or by directly engaging with gut associated immune cells, HMOs influence the development and function of the host immune defenses. HMOs elicit expression of inflammatory cytokines and chemokines in gut epithelial cells which allows for the recruitment of innate immune cells. HT-29 colonic epithelial cells were shown to produce a variety of cytokines; such as IL-8, IL-1b, and IL-17C and chemokines; CXCL1, CXCL3, CCL20 (52), all of which are key chemoattractants and inflammatory cytokines with the ability to activate a range of immune cells. It is thought that this mechanism occurs to prevent prevalence of pathogenic bacterial colonization in the gut through priming innate immune cells. HMOs can also enhance mucosal immunity by inducing antimicrobial peptides, secretory IgA antibodies (136), and other immune factors that contribute to mucosal defense mechanisms. This helps bolster the innate and adaptive immune responses against infections at primary pathogen contact points in the body.

#### 3.3.1 Effects of human milk oligosaccharides on the immune system through modulation of the microbiome

These complex sugars facilitate microbial diversity in the infant gut by nurturing various beneficial bacterial species (137). A diverse gut microbiome correlates with improved overall health outcomes, including better nutrient absorption (138), bolstered immune function, and defence against pathogens (139). Furthermore, HMOs are recognized for their role in maintaining the integrity of the gut barrier by promoting mucin production (140, 141), which is essential for preventing harmful bacteria and toxins from crossing the gut lumen into the bloodstream.

HMOs act as a nutritional source for specific beneficial bacteria, such as bifidobacteria and lactobacilli, in the infant gut. These bacteria possess glycosidase enzymes capable of breaking down and fermenting HMOs, enabling them to thrive and dominate the gut microbial community (142, 143). Among these, bifidobacteria stand out for their adeptness at utilising HMOs as a fermentation substrate (144). Consequently, breastfed infants who receive HMOs through breast milk typically harbour a higher abundance of bifidobacteria in their gut microbiome compared to their formula-fed counterparts. This selective enrichment of bifidobacteria contributes to a more favourable microbial composition overall.

Short-chain fatty acids (SCFAs) also arise from the microbial fermentation of indigestible dietary components within the intestinal tract, serving as an energy source for colonocytes (145). HMOs are selectively fermented by commensal bacteria, such as bifidobacteria, as well as lactobacilli (146), and *Anaerobutyricum hallii* (147), which positively impact the gut microbiome (148), and produce SCFAs (149). Upon absorption by colonocytes, SCFAs engage with G protein-coupled receptors (GPCRs) (150) through their interaction with specific receptors and enzymatic pathways, can have a positive impact on the gut barrier, reduce inflammation, and enhance immune function (151). SCFAs are involved in the metabolism which regulates obesity, type-2-diabetes and insulin resistance (152) therefore their production through HMO fermentation in the gut plays a role in the outcome of these diseases. Additionally, SCFAs modulate histone deacetylases (HDACs) (153), further influencing intestinal mucosal immunity, barrier integrity, and cellular function. Acetate (C2), being convertible to Acetyl-CoA, is a crucial substrate in protein acetylation reactions and can directly influence cellular signalling. Enhanced acetate levels promote the acetylation of glyceraldehyde 3-phosphate dehydrogenase (GAPDH) and augment glycolytic pathways (154), which in turn can give rise to Th1 CD4^+^ T-cells. Peripherally, SCFAs influence systemic inflammation mainly by inducing Treg differentiation and by regulating the secretion of interleukins (155–157). These intricate interactions emphasise the pivotal role of HMO-induced SCFAs in orchestrating diverse cellular processes crucial for maintaining intestinal homeostasis and immune function.

#### 3.3.2 Protective properties of human milk oligosaccharides

Studies have shown that HMOs may impact the severity and duration of infections, potentially reducing the intensity of symptoms and hastening recovery. The dual role of HMOs in both preventing and mitigating infections shows their significance in promoting the health and resilience of individuals, particularly during infancy when the immune system is still developing (158–160).

##### 3.3.2.1 Antimicrobial and antiviral activity

Certain HMOs can directly inhibit the growth of pathogens. Various HMOs were found to significantly inhibit the growth of three strains of Group B *Streptococcus* (GBS), indicating antimicrobial activity. This was determined by comparing bacterial biomass in the presence and absence of HMOs, showing significant inhibition across different growth conditions (161).

HMOs can also enhance the effectiveness of antibiotics against MDR bacteria by lowering the minimum inhibitory concentration (MIC) of various antibiotics (gentamicin, erythromycin, clindamycin and minocycline) by a significant amount against GBS (162). Furthermore, HMOs can boost the potency of trimethoprim, an antifolate antibiotic, resulting in a substantial decrease in MIC against GBS compared to the antibiotic alone (163).

In the case of viral infection; HMOs, 2′FL, LNnT, 6′SL and 3′SL, were studied *in vitro* for their ability to prevent influenza and RSV infections (164). Results showed that 6′SL and LNnT pre-treatments for 24-hours reduced influenza replication in 16HBE respiratory epithelial cells. RSV protection was shown with 2′FL pre-treatments in both 16HBE and Calu-3 cell lines, but 3′SL pre-treatments only protected in the 16HBE cells. This pre-treatment of respiratory epithelial cells with HMOs may act to strengthen the structural integrity of cell-cell adhesion, similar to what HMOs do in the gut to prevent gastrointestinal bacterial infections (97, 118, 165).

##### 3.3.2.2 Anti-biofilm activity

Recent research has revealed that HMOs have antimicrobial and antibiofilm effects against gram-positive bacteria, particularly against *Enterococcus faecalis* and *E. faecium* in their planktonic forms, and also reduce *S. aureus* biofilm formation (166). These findings align with previous research indicating the effectiveness of HMOs against primarily Gram-positive bacteria with Ackerman et al. demonstrating biofilm reduction in *Streptococcus agalactiae* and *S. aureus* by 93% and 60% respectively (161) corroborated by other studies (167). This inhibition of biofilm formation by HMOs may contribute to reducing the virulence and pathogenicity of such bacteria. *In vitro* experiments utilizing 3’-SL and 6’-SL resulted in the reduced adhesion of *Clostridium difficile* to human colon cells. Additionally, they showed a downregulation of the *cwp84* gene (168), encoding the Cwp84 protein, a key virulence factor of *C. difficile* which is involved in biofilm formation (169).

As well as preventing biofilm formation, HMOs may also disrupt the structure of biofilm matrices formed by pathogenic bacteria, making them more susceptible to antimicrobial agents or immune defenses. For example, research demonstrated that HMOs can interfere with the biofilm matrix of GBS (170), a common neonatal pathogen. This disruption of biofilm integrity may enhance the efficacy of antimicrobial treatments against GBS infections.

##### 3.3.2.3 Decoy receptors and competitive inhibition

HMOs possess anti-adhesive properties, in which they inhibit bacterial attachment, a pivotal step in pathogen establishment at mucosal surfaces throughout the body, including the gut and respiratory tract (171). They do this by acting as decoy receptors, through competitively binding to PAMPs, preventing their adhesion to host cell receptors and subsequent invasion into epithelial cells. This interference disrupts the pathogen’s ability to establish infection, thereby conferring protection to the host. HMOs are capable of preventing the attachment of many pathogenic bacteria such as *Escherichia coli* (172), *Vibrio cholerae*, *Salmonella fyris* (173), *Campylobacter jejun*i (174), *Helicobacter pylori* (175, 176), and *Streptococcus pneumoniae* (177), or viruses including Human Rotavirus (178) and Parainfluenza Virus (SV5) (179), among others (180). The pathogens bind to HMOs which heavily resemble glycan receptors that these pathogens target on the surface of epithelial cells. For example, 2′-FL can mimic the norovirus receptor, blood group antigen (HBGA), serving as a decoy to inhibit the virus attaching to its receptor; HBGA (181). *In vitro* studies have demonstrated that 2′-FL and 3-FL inhibited the infection of Vero E6 cells by SARS-CoV-2 pseudoviruses. Both HMOs were most effective against the Omicron variant. This suggests that 2′-FL and 3-FL have potential antiviral activity against SARS-CoV-2, by interfering with viral receptor binding (182).

In a similar fashion of preventing pathogenic uptake into epithelial cells via binding glycan receptors by binding to the pathogens, HMOs can bind to the glycan receptors themselves, also preventing pathogenic attachment and establishment within the epithelia.

## 4 Clinical implications of human milk oligosaccharides

The mechanistic insights provided in the previous sections highlight the therapeutic potential of HMOs in clinical settings. By targeting specific immune receptors such as TLRs and Siglecs (Section 3.1), HMOs could be used to design therapies for modulating immune responses. The ability of HMOs to enhance or suppress immune cell functions (Section 3.2) suggests their utility in treating conditions such as inflammatory bowel disease, infections, or autoimmune disorders. Furthermore, the systemic effects of HMOs on gut microbiota and overall immune homeostasis (Section 3.3) underscore their role in supporting immune resilience in neonates and adults alike. Understanding the clinical implications of HMOs on immune system modulation is crucial for developing therapeutic interventions and nutritional strategies for various health conditions. HMOs have shown promise in clinical settings for managing allergic diseases and potentially serving as therapeutic agents in various immune-related conditions.

### 4.1 Management of allergic diseases

The potential role of oligosaccharides in allergy has been widely discussed (183–188). HMOs have been found to reduce the incidence of allergies and eczema in infants (186, 189). Multiple trials studying the effects of prebiotics on allergy prevention highlight the potential role of HMOs and other prebiotic oligosaccharides in mitigating allergic reactions (190, 191).

#### 4.1.1 Eczema (atopic dermatitis)

Atopic dermatitis, or eczema, is a chronic disease that causes inflammation and irritation of the skin. Clinical studies have suggested that young infants exposed to HMOs, such as 2’-FL or LNFP-I, may have reduced risk of eczema development (192). Infants who are fed breast milk high in HMOs tend to experience a lower occurrence of eczema when compared to those who are given formula. Some studies analyzing the effects of bifidobacteria on atopic dermatitis symptoms shows that the prevalence of this bacteria was associated with reduction of allergy conditions (193–195), therefore HMOs, may exert their protective effects by promoting bifidobacteria colonization in the gut in addition to modulating immune responses and promoting immune tolerance.

#### 4.1.2 Asthma

Asthma is a chronic lung disease caused by inflammation and muscle tightening around the airways. While research is ongoing, preliminary studies suggest that HMOs may play a role in reducing the risk of asthma development in infants (196). HMO supplementation in early life in a murine model has been associated with reduced lung histopathology scores, circulating IgE, cytokine levels, and inflammatory cell infiltration (197). The results of this study also demonstrated an increase of SCFAs in the gut and blood of treated mice. The gut microbiota-generated SCFA acetate (formed through selective fermentation of HMOs) led to offspring being more resistant to asthma later in life (198). With other SCFAs butyrate and propionate separately regulating DC function in turn managing T-cell responses, generating a tolerogenic immune response and enhanced Th1 phenotype which is protective against asthma development, as asthma is exacerbated predominantly by a Th2 phenotype response (199–201).

#### 4.1.3 Food allergies (atopy)

Atopy is typically associated with heightened immune responses to common allergens, especially inhaled allergens and food allergens. HMOs have been investigated for their potential to prevent the development of food allergies in infants (189). Studies have shown that breastfed infants, who naturally receive HMOs, have a lower risk of developing food allergies compared to formula-fed infants (202). The specific modulation of cytokine output by HMOs in cells closely linked to allergy also insinuates that HMOs may play a role in food allergy (120). HMOs have also recently be shown to modulate Antigen-IgE complex activation of human epithelial cells that may have important implications for food-allergy (203). Additionally an attenuation in food allergy symptoms in a mouse model has been observed with mice that had daily oral treatment of 2’-FL and 6’-SL (114). Other studies employing mouse models found that 2’-FL specifically attenuates β-LG induced food allergy inflammatory symptoms via TLR4/NFκB signaling as 2’-FL itself was shown to reduce expression of key downstream proteins of the TLR4 signaling pathway. 2’-FL also induced increased expression of miR-146a, a micro RNA, which has been shown to also inhibit TLR4 signaling (80). HMOs may influence immune responses in the gut and promote immune tolerance to food antigens in this manner, reducing the likelihood of allergic sensitization.

### 4.2 Autoimmune and inflammatory diseases

Clinical trials are underway to evaluate the safety and efficacy of HMO supplementation in autoimmune diseases. These trials aim to elucidate the mechanisms of action of HMOs in autoimmune pathogenesis and determine their potential as adjunctive or alternative therapies for autoimmune diseases due to their immunomodulatory properties. Studies to date show that the HMOs are well tolerated and safe for both infant and adult populations (204, 205).

#### 4.2.1 Necrotizing enterocolitis

Necrotizing Enterocolitis (NEC) is a severe gastrointestinal condition primarily affecting premature infants, characterized by inflammation and necrosis of the intestinal mucosa (206). The involvement of HMOs in NEC prevention has been previously postulated (207–209). In general HMO supplemented NEC patients were observed to have lower pathology scores (210, 211) and also reduced cases of NEC (204). HMOs have been shown to reduce the risk of NEC development by promoting the growth of beneficial gut bacteria, enhancing gut barrier function, and modulating inflammatory responses. These protective effects contribute to the overall reduction of infection-related morbidity and mortality in vulnerable neonates.

Numerous studies have identified disialyllacto-*N*-tetraose (DSLNT) as being protective in the context of NEC (212). In a mast cell activation *in vitro* model, DSLNT suppressed certain inflammatory markers released by mast cells including histamine. In the same study using an *in vivo* model, newborn rats fed DSLNT-supplemented formula displayed reduced incidence and severity of NEC, with lower inflammatory markers and cytokines in the gut (115). Clinically, low DSLNT levels were actually associated with NEC development in infants (213, 214), suggesting a potential diagnostic marker. Additionally, the levels of lacto-*N*-difucohexaose I (LNDFH-I) were lower in breast milk samples from mothers associated with NEC cases, as compared to those associated with non-NEC cases. In general, a lack of HMO diversity can be associated with a higher incidence rate of NEC in infants (215).

Sialylated HMO treatment reduced mast cell accumulation and suppressed inflammatory markers in the intestines of NEC rats. *In vitro*, using colon epithelial cells; Caco-2, HMOs protected cells from damage induced by various agents and regulated cell cycle phases, enhancing cell viability (116). HMOs decreased NEC intestinal injury and boosted proliferation and stem cell activity in reduced microbiota conditions. In germ-free conditions, HMOs stimulated intestinal organoid growth (216). Administration of 2′FL protected against NEC in mice by preserving intestinal architecture and enhancing perfusion through increased endothelial nitric oxide synthase (eNOS) expression, linking HMOs to improved gut health (217).

HMO administration reduces inflammation and promotes intestinal cell proliferation in NEC models. HMOs inhibit TLR4 signaling, enhancing enterocyte growth and maturation (218, 219). Specific HMOs such as 2’-FL and 6’-SL reduce NEC incidence by targeting TLR4-mediated inflammation, supported by molecular docking studies (220). HMOs alter gene expression related to cell differentiation (115) and signaling (219), defending against NEC by preventing damage to IECs and promoting intestinal barrier function through MUC2 differentiation (221). MUC2 (mucin 2), is a type of mucin, which is a family of high molecular weight, heavily glycosylated proteins produced by epithelial tissues in the mucosa. MUC2 is specifically the major gel-forming mucin found in the mucus that lines the gut, particularly in the large intestine (222). This enables MUC2 to assist the immune system by inhibiting pathogen adhesion to the epithelial cells, thereby preventing their entry. Consequently, the promotion of MUC2 would be considered beneficial for preventing NEC in infants. Supplementation with the HMO DSLNT reduces NEC severity in formula-hand-fed rats by modulating inflammatory markers and cellular pathways, such as apoptosis (210) thereby improving intestinal health.

#### 4.2.2 Inflammatory bowel disease

Inflammatory bowel disease (IBD) is a term for two conditions ((Crohn’s disease (CD) and ulcerative colitis (UC)) which are characterized by chronic inflammation of the gastrointestinal (GI) tract (223, 224). HMOs have emerged as potential therapeutic agents for inflammatory bowel diseases (225, 226). Preclinical studies using Caco-2 cell monolayers have demonstrated that sialylated HMOs; 3’-SL and 6’-SL possess anti-inflammatory properties and can help maintain gut barrier integrity (165). Clinical trials are underway to evaluate the efficacy of HMO supplementation in managing IBD symptoms and reducing inflammation, with some showing an alleviation of symptoms (227–230). HMOs have shown potential in reducing inflammation and improving gut barrier function in animal models of IBD. Clinical trials are required to evaluate the efficacy of HMO supplementation in managing IBD symptoms and reducing disease activity in humans.

#### 4.2.3 Type 1 diabetes mellitus

Type 1 diabetes (T1D) is a T-cell mediated autoimmune disease in which destruction of pancreatic β-cells causes insulin deficiency which leads to hyperglycaemia and a tendency to ketoacidosis (231). HMOs may aid with a T1D prognosis as they increase the abundance of *B*. *infantis*, the presence of which has been observed to prevent autoimmune diabetes due to the associated increase in SCFA production (232–234). HMOs can therefore be indirectly linked to the decrease in T1D incidences in breast fed infants due to their induction of SCFA metabolites (235). Furthermore, exposure to HMOs in early life has been demonstrated to inhibit the onset of T1D in NOD mice, as well as lessen the severity of pancreatic insulitis later in life (106).

#### 4.2.4 Rheumatoid arthritis

Rheumatoid arthritis (RA) is a chronic disease that causes inflammation around the body mainly in the joints (236). Animal studies have demonstrated that HMO supplementation can attenuate joint inflammation and reduce disease severity in models of RA, specifically collagen-induced arthritis (CIA), a widely used autoimmune model of RA. This effect is achieved by completely inhibiting the phosphorylation of p65, a key protein involved in the NFκB signaling pathway (237) thereby subverting the inflammatory response and contributing to decreased joint destruction and overall disease progression. A mini-pig RA model where 3’-SL was directly injected into the affected joint showed an improvement of pathological symptoms at a cellular level, however the researchers found no difference in inflammatory markers between the test and control (238). The authors suggest that this effect may be due to the direct injection of 3’-SL into the joint, as opposed to oral administration used in other studies analyzing the effects of HMOs in different inflammatory disease models. Both of these studies suggest that 3’-SL directly does not exert a protective effect against RA but rather has an ancillary role through other effects on gut bacteria and the immune system.

#### 4.2.5 Multiple sclerosis

Multiple Sclerosis (MS) is a chronic autoimmune disorder in which sheaths of the central nervous system are damaged and inflamed (239). Preclinical studies have suggested that HMOs may modulate immune responses and reduce inflammation *ex vivo*. 2’-FL and LNFP-III were shown to decrease MNC proliferation and production of inflammatory cytokines in samples from MS patients (96). As monocytes play a key role in MS development, even in early stages of MS (240), this study may suggest a certain therapeutic effect of HMOs in MS but also potentially other monocyte-mediated diseases.

## 5 Discussion

The intricate interactions of HMOs with the immune system highlight their multifaceted roles in modulating immune responses and promoting health. A significant aspect of HMO function lies in their impact on gut-associated immune responses. HMOs indirectly modulate the immune system by shaping the gut microbiome, fostering a beneficial microbial environment that supports immune cell homeostasis. Moreover, HMOs exhibit protective properties, offering defense against pathogens and reducing the risk of infections. However HMOs directly show potent immunomodulatory effects, and elucidating how these bioactive compounds influence immune cell behavior and developmental immunomodulation is warranted. HMOs engage with specific receptors on immune cells, leading to diverse responses that are critical during the early stages of immune system development.

The clinical implications of HMOs are profound, particularly in managing allergic diseases, where they have been shown to reduce the incidence and severity of allergic reactions. Their potential in addressing autoimmune and inflammatory diseases also presents a promising avenue for therapeutic intervention. HMOs have immunomodulatory effects beyond infancy and their therapeutic applications are expanding, with ongoing research exploring their efficacy in various clinical settings. Although further clinical studies are needed to elucidate the mechanisms of action and optimize the therapeutic potential of HMOs in these contexts.

**Figure 7:**
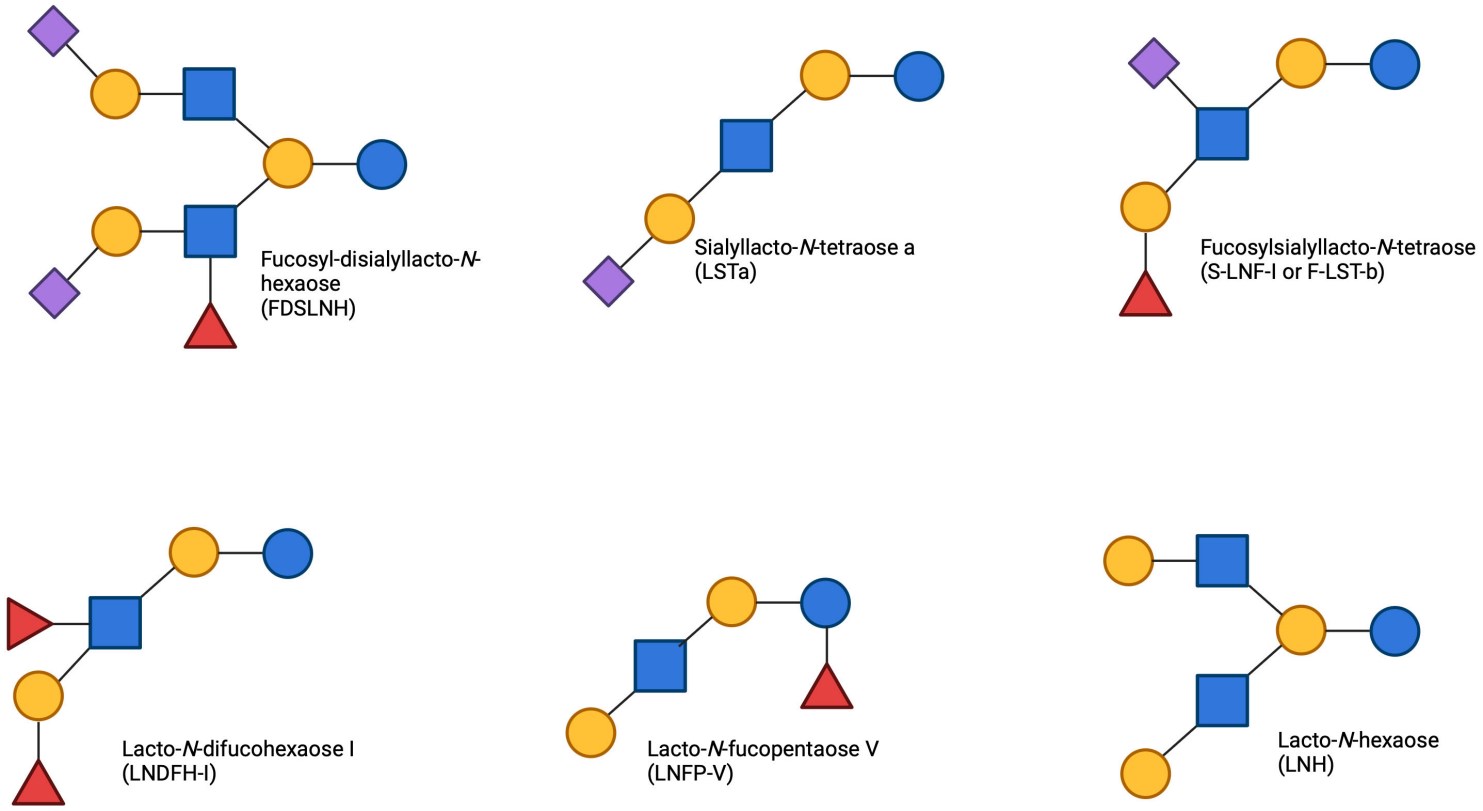
Uncommon HMOs found in human breast milk The less prevalent HMOs found in breast milk, display incredible structural diversity. Featured HMOs include fucosyl-disialyllacto-*N*-hexaose (FDSLNH), sialyllacto-*N*-tetraose a (LSTa), sialyl-lacto-*N*-fucopentaose I (S-L*N*F-I), lacto-*N*-difucohexaose I (L*N*DFH-I), lacto-*N*-fucopentaose V (LNFP-V), and lacto-*N*-hexaose (LNH). The unique structures of these HMOs emphasizes their varied presence and potential functional contributions within human milk.

The most commonly found HMOs, namely 2’-FL, 3’-SL, and LNT, have been extensively studied for their health benefits. Despite the significant insights gained from these common HMOs, the less commonly found HMOs, such as lacto-*N*-hexaose (LNH) and disialyllacto-*N*-tetraose II (DSLNT II), remain underexplored (**Figure 7**). One major reason for this is the difficulty in isolating and producing these uncommon HMOs commercially. Their low abundance in human milk and the complexity of their structures make it challenging to obtain sufficient quantities for detailed studies. Given the structural diversity and unique properties of these uncommon HMOs, there is a compelling potential for them to exhibit notable immunological effects. Research into these less abundant HMOs could uncover new mechanisms by which they influence the immune system, offering insights into additional ways that human milk supports infant health and potentially leading to novel therapeutic applications.

In summary, the comprehensive immunomodulatory effects of HMOs highlight their importance in early life nutrition and potential therapeutic applications. Their ability to interact with the immune system, modulate the microbiome, and provide protective benefits positions HMOs as crucial components in promoting health and preventing disease. While the benefits of HMOs in the gut are clear, there is still much to learn about these complex sugars. Further research is needed to fully understand the mechanisms by which HMOs promote the growth of beneficial gut microbes. Additionally, further research is needed to understand how HMOs interact with immune cells and how they alter their function and fate. Furthermore, we still need to determine the mechanisms by which HMOs promote these aforementioned health outcomes and to identify the specific types of HMOs that are most beneficial for different populations based on potential bacterial threats to specific countries and regions. This could lead to specified therapeutic options using HMOs as a prebiotic, not only promoting beneficial bacteria, but preventing pathogenic infection. Further research is essential to fully harness the therapeutic potential of HMOs, paving the way for innovative treatments that leverage their unique properties.
